# Heat Shock Transcription Factor σ^32^ Co-opts the Signal Recognition Particle to Regulate Protein Homeostasis in *E. coli*

**DOI:** 10.1371/journal.pbio.1001735

**Published:** 2013-12-17

**Authors:** Bentley Lim, Ryoji Miyazaki, Saskia Neher, Deborah A. Siegele, Koreaki Ito, Peter Walter, Yoshinori Akiyama, Takashi Yura, Carol A. Gross

**Affiliations:** 1 Department of Microbiology and Immunology, University of California at San Francisco, San Francisco, California, United States of America; 2 Institute for Virus Research, Kyoto University, Kyoto, Japan; 3 Department of Biochemistry and Biophysics and Howard Hughes Medical Institute, University of California at San Francisco, San Francisco, California United States of America; 4 Department of Biology, Texas A&M University, College Station, Texas, United States of America; 5 Faculty of Life Sciences, Kyoto Sangyo University, Kyoto, Japan; 6 Department of Cell and Tissue Biology, University of California at San Francisco, San Francisco, California, United States; Yale School of Medicine, United States of America

## Abstract

All cells must adapt to rapidly changing conditions. The heat shock response (HSR) is an intracellular signaling pathway that maintains proteostasis (protein folding homeostasis), a process critical for survival in all organisms exposed to heat stress or other conditions that alter the folding of the proteome. Yet despite decades of study, the circuitry described for responding to altered protein status in the best-studied bacterium, *E. coli*, does not faithfully recapitulate the range of cellular responses in response to this stress. Here, we report the discovery of the missing link. Surprisingly, we found that σ^32^, the central transcription factor driving the HSR, must be localized to the membrane rather than dispersed in the cytoplasm as previously assumed. Genetic analyses indicate that σ^32^ localization results from a protein targeting reaction facilitated by the signal recognition particle (SRP) and its receptor (SR), which together comprise a conserved protein targeting machine and mediate the cotranslational targeting of inner membrane proteins to the membrane. SRP interacts with σ^32^ directly and transports it to the inner membrane. Our results show that σ^32^ must be membrane-associated to be properly regulated in response to the protein folding status in the cell, explaining how the HSR integrates information from both the cytoplasm and bacterial cell membrane.

## Abstract 2

The bacterial heat shock transcription factor, Ïƒ32, maintains proper protein homeostasis only after it is targeted to the inner membrane by the signal recognition particle (SRP), thereby enabling integration of protein folding information from both the cytoplasm and cell membrane.

## Author Summary

All cells have to adjust to frequent changes in their environmental conditions. The heat shock response is a signaling pathway critical for survival of all organisms exposed to elevated temperatures. Under such conditions, the heat shock response maintains enzymes and other proteins in a properly folded state. The mechanisms for sensing temperature and the subsequent induction of the appropriate transcriptional response have been extensively studied. Prior to this work, however, the circuitry described in the best studied bacterium *E. coli* could not fully explain the range of cellular responses that are observed following heat shock. We report the discovery of this missing link. Surprisingly, we find that σ^32^, a transcription factor that induces gene expression during heat shock, needs to be localized to the membrane, rather than being active as a soluble cytoplasmic protein as previously thought. We show that, equally surprisingly, σ^32^ is targeted to the membrane by the signal recognition particle (SRP) and its receptor (SR). SRP and SR constitute a conserved protein targeting machine that normally only operates on membrane and periplasmic proteins that contain identifiable signal sequences. Intriguingly, σ^32^ does not have any canonical signal sequence for export or membrane-integration. Our results indicate that membrane-associated σ^32^, not soluble cytoplasmic σ^32^, is the preferred target of regulatory control in response to heat shock. Our new model thus explains how protein folding status from both the cytoplasm and bacterial cell membrane can be integrated to control the heat shock response.

## Introduction

The heat shock response (HSR) maintains protein homeostasis (proteostasis) in all organisms. The HSR responds to protein unfolding, aggregation, and damage by the rapid and transient production of heat shock proteins (HSPs) and by triggering other cellular protective pathways that help mitigate the stress. Although the specific HSR is tailored to each organism, chaperones that mediate protein folding and proteases that degrade misfolded proteins are almost always included in the core repertoire of induced protein and are among the most conserved proteins in the cell. These HSPs maintain optimal states of protein folding and turnover during normal growth, while decreasing cellular damage from stress-induced protein misfolding and aggregation. Malfunction of the HSR pathway reduces lifespan and is implicated in the onset of neurodegenerative diseases in higher organisms [1]–[3].

**Figure 1:**
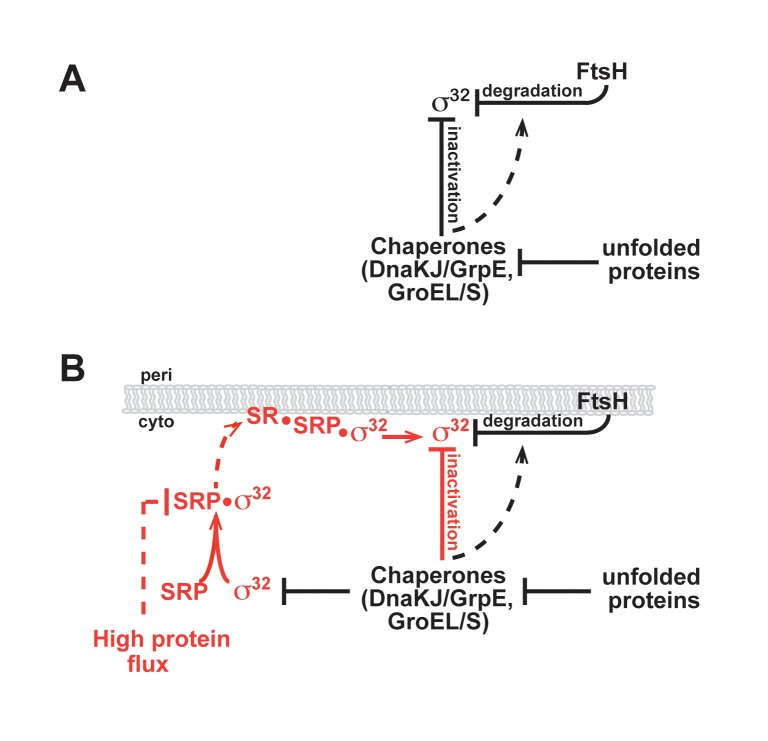
Homeostatic control circuits of σ^32^. (A) Current and (B) revised model for activity and degradation control of σ^32^. The revised model incorporates SRP-mediated trafficking of σ^32^ to the membrane. Interactions validated *in vitro* are shown as solid lines; those inferred from *in vivo* data are shown as dashed lines. Newly identified interactions are shown in red.

In *E. coli* and other proteobacteria, σ^32^ mediates the HSR by directing RNA polymerase to promoters of HSR target genes [4]–[9]. Given the importance of this response and the necessity for a rapid but transient increase in expression of HSPs, it is not surprising that regulation of the HSR across organisms is complex. σ^32^ is positively regulated by a feed-forward mechanism in which exposure to heat melts an inhibitory mRNA structure enabling high translation of σ^32^ mRNA [10],[11] and is negatively regulated by two feedback loops [12] mediated through members of the σ^32^ regulon (Figure 1A). σ^32^ activity is coupled to the cellular protein folding state via a negative feedback loop executed by the two major chaperone systems, DnaK/J/GrpE and GroEL/S. There is extensive support for the model that free chaperones directly inactivate σ^32^ and that these chaperones are titrated by unfolded proteins that accumulate and bind chaperones during a HSR. Depletion of either chaperone system or overexpression of chaperone substrates leads to an increase in σ^32^ activity, and conversely, overexpression of either chaperone system decreases σ^32^ activity [13],[14]. Inhibition is likely direct, as DnaK/J and GroEL/S bind σ^32^ *in vitro* and inhibit its activity in a purified *in vitro* transcription system [13],[15]–[17]. σ^32^ stability is controlled by the inner membrane (IM) protease FtsH: deletion of the protease stabilizes σ^32^ [18]–[20], and FtsH degrades σ^32^ *in vitro*, albeit slowly [18],[20]. DnaK/J and GroEL/S also regulate stability, as their depletion leads to σ^32^ stabilization *in vivo* [13],[14],[21], although this finding has not yet been recapitulated *in vitro* [22].

Despite the regulatory complexity of the current model, it inadequately addresses two issues that are central to our understanding of the circuitry controlling the HSR, motivating us to search for additional players in the response: (1) Exhaustive genetic screens for mutations in σ^32^ that result in misregulation have identified a small cluster of four closely spaced amino acid residues (Leu47, Ala50, Lys51, and Ile54), of which three are surface exposed, as well as a somewhat distant fifth residue that abuts this patch in the folded σ^32^ structure. When these residues are mutated, cells have both increased level and activity of σ^32^, indicating that this region is involved in a central process required for operation of the negative feedback loops that control both the activity and degradation of σ^32^ (Figure 1A) [23]–[25]. However, the phenotypes of these mutants are not recapitulated *in vitro*, where both FtsH degradation and chaperone-mediated inactivation of mutant and WT σ^32^ are experimentally indistinguishable [25],[26]. Thus, we do not understand how this “homeostatic control region” of σ^32^ functions. (2) σ^32^ is thought to monitor the folding status of IM proteins as well as cytoplasmic proteins, but the mechanism for this additional surveillance is unknown. Their close connection is indicated because (1) the IM protease, FtsH, not only degrades σ^32^, but also maintains quality control in the IM by degrading unassembled IM proteins; (2) induction of the HSR is a very early response to perturbations in the co-translational membrane-trafficking system that brings ribosomes translating IM proteins to the membrane [27]–[29]; and (3) IM proteins are significantly overrepresented both in the σ^32^ regulon [30] and in an unbiased overexpression screen for HSR inducers [30].

**Figure 2:**
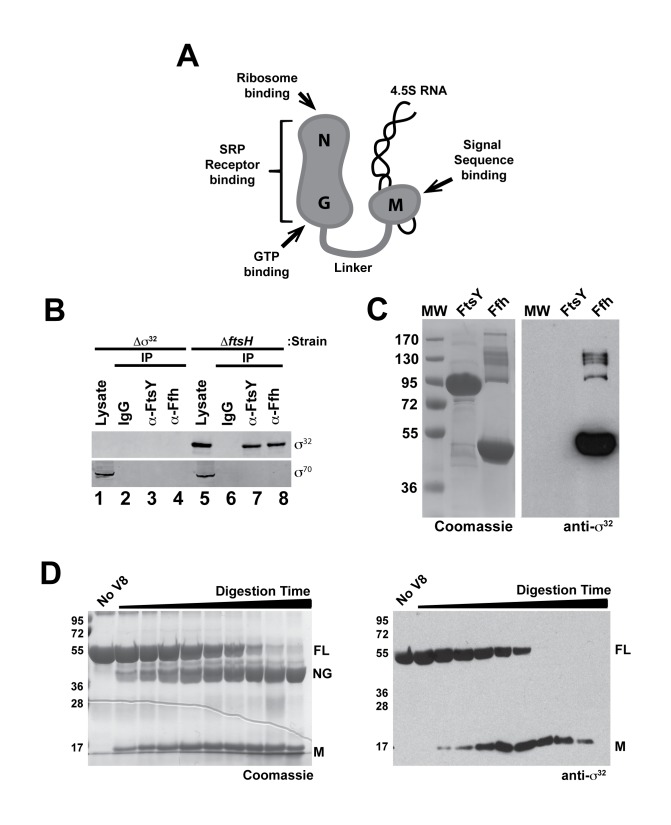
σ^32^ binds to Ffh. (A) Schematic representation of *E. coli* SRP (Ffh+4.5S RNA), indicating experimentally confirmed functions associated with each domain. (B) σ^32^ co-immunoprecipitates with Ffh and FtsY *in vivo*, but σ^70^ does not. Immunoprecipitations of Ffh or FtsY were carried out on lysates of Δσ^32^ and Δ*ftsH* cells grown to exponential phase. Immunocomplexes were isolated, analyzed by SDS-PAGE, and immunoblotted with anti-σ^32^ and anti-σ^70^ antibodies. Proteins from approximately 15-fold more cells were loaded onto the gel for the immunoprecipitated samples against σ^32^ and σ^70^ as compared with the lysate samples. (C) Protein–protein interaction analysis indicates that σ^32^ binds to Ffh, but not FtsY. Purified FtsY and Ffh were run on a 10% SDS-PAGE gel, transferred to nitrocellulose, re-natured, and incubated with purified WTσ^32^. The Coomassie-stained gel (left) and the nitrocellulose blot probed with polyclonal anti-σ^32^ antibodies (right) are shown. (D) σ^32^ binds to the M-domain of Ffh. Ffh, partially digested by endopeptidase V8, was resolved on a 10% SDS-PAGE gel, transferred to nitrocellulose, and incubated with σ^32^. The Coomassie-stained gel (left) and the nitrocellulose membrane, containing transferred Ffh fragments, probed against σ^32^ (right) are shown.

In this report, we identify the co-translational protein targeting machinery, comprised of the Signal Recognition Particle (SRP; Ffh protein in complex with 4.5S RNA; Figure 2A) and the SRP Receptor (SR; FtsY), as a regulator of σ^32^. We show that SRP preferentially binds to WTσ^32^ compared to a mutant σ^32^ with a defective homeostatic control region. We further show that a fraction of σ^32^ is associated with the cell membrane and that both the SRP-dependent machinery and the homeostatic control region of σ^32^ are important for this localization. Lastly, the regulatory defects in HSR circuitry caused by mutation of either the σ^32^ homeostatic control region or the co-translational targeting machinery are circumvented by artificially tethering σ^32^ to the IM. We propose that SRP-dependent membrane localization is a critical step in the control circuitry that governs the activity and stability of σ^32^. Membrane localization is widely used to control σ factors, but this is the first case where the IM-localized state is used for dynamic regulation rather than as a repository for an inactive protein.

## Results

### A Transposon Insertion Mutant at the *ftsY* Promoter Region Is Defective in Feedback Control

To identify additional players involved in activity control of σ^32^, we carried out a genetic screen for transposon mutants with increased σ^32^ activity under conditions that inactivate σ^32^ in wild-type cells (see Methods). To impose a condition that mimics the negative feedback control of σ^32^, the DnaK/J chaperones were overexpressed from an inducible promoter at their chromosomal locus. Under these conditions, a σ^32^-regulated *lacZ* chromosomal reporter (P*_htpG_*-*lacZ*) is expressed so poorly that cells do not make sufficient β-galactosidase to turn colonies blue on X-gal indicator plates. We screened for blue colonies, indicative of a defect in σ^32^ inactivation. A conceptually similar screen previously identified mutations in the DnaK/J chaperones—key negative regulators of the σ^32^ response [31]. In addition to re-identifying these components, we found an insertion in the promoter region of *ftsY* (p*ftsY*::Tn*5*), located 39 bp upstream of the *ftsY* open reading frame. The p*ftsY*::Tn*5* strain had a 3- to 4-fold reduction in the level of FtsY, the SR, and a ∼7-fold increase in the activity and amount of σ^32^ relative to WT (Table 1). Defects were complemented by a plasmid carrying *ftsY*. Unlike WT, in the p*ftsY*::Tn*5* strain σ^32^ activity did not respond to increased chaperone expression. Upon chaperone overexpression in WT cells, the specific activity (S.A.) of σ^32^ fell to 0.3, relative to that in cells growing without chaperone overexpression. In contrast, upon chaperone overexpression in p*ftsY*::Tn*5* cells, the S.A. of σ^32^ did not change, suggesting a defect in chaperone-mediated activity control in that strain (Table 1). This finding raised the possibility that the high activity of σ^32^ in p*ftsY*::Tn*5* resulted from disruption of activity control of σ^32^, rather than reflecting a cellular response to accumulation of unassembled membrane proteins.

**Table 1:** The altered σ^32^ phenotypes of the Tn5 insertion mutant (p*ftsY*::Tn5) are significantly complemented by an *ftsY* ^+^ plasmid.

				Relative S.A. of σ^32^	
				Chaperone Overexpression	
Strain	FtsY Level	σ^32^ Level	σ^32^ Activity	None	+DnaKJ/GrpE
Wild-type	1.0±0.2	1.0±0.1	1.0±0.1	1.0	0.3
p*ftsY*::Tn5	0.3±0.1	6.8±0.6	7.7±1.0	1.1	1.1
p*ftsY*::Tn5 +*pftsY* ^+^ *	0.9±0.1	2.5±0.4	2.1±0.5	0.8	0.3

In this and all other experiments, protein levels were determined by SDS-PAGE followed by quantitative immunoblotting. σ^32^ activity was determined from a chromosomal β-galactosidase reporter (calculated as a differential rate of synthesis); values presented are from ≥3 experiments. Relative S.A. of σ^32^ is defined as: [(σ^32^ activity/σ^32^ level) normalized to σ^32^ S.A. of WT cells grown at 30°C].

* The *ftsY* ^+^ plasmid inhibits growth of the cells by ∼30%.

### σ^32^ Directly Interacts with SRP

We tested whether σ^32^ binds to either FtsY (SR) or to Ffh, the protein component of SRP. Ffh is a two-domain protein, comprised of an M-domain that binds the signal sequence and 4.5S RNA, and an NG-domain that binds to SR, the ribosome, and GTP (Figure 2A). We first used co-immunoprecipitation analysis. Interacting proteins were immunoprecipitated with antibodies against either FtsY or Ffh and, following resolution on SDS-PAGE, antibodies against σ^32^ or σ^70^ were used to probe for the presence of these proteins. σ^32^ was detected in the immunoprecipitations (Figure 2B, lanes 7 and 8), and this signal was dependent on the presence of σ^32^ in the strain (Figure 2B, lanes 1–4). By contrast, σ^70^, although much more abundant than σ^32^ in the cell, did not interact with either SRP or SR (Figure 2B, Lanes 3,4 and 7,8), indicating that interaction with SRP is not a general property of σs. It was not surprising that σ^32^ was co-immunoprecipitated with both SRP and SR, as the latter two components interact *in vivo*. To determine the direct binding partner of σ^32^, purified Ffh and FtsY were resolved on SDS-PAGE, transferred to nitrocellulose, and incubated with purified σ^32^. Antibodies against σ^32^ detected σ^32^ present at the molecular weight corresponding to Ffh but not SR (Figure 2C). In a reciprocal experiment, purified σ^32^ was resolved on SDS-PAGE, transferred to nitrocellulose, and incubated with purified Ffh or SR. Ffh, but not SR, bound σ^32^ (unpublished data). Similar studies did not reveal an interaction between σ^70^ and either Ffh or SR (unpublished data). We determined which Ffh domain binds σ^32^ by partially-proteolyzing Ffh to produce an 18 kDa M-domain and a 38 kDa NG-domain, resolving the mixture by SDS-PAGE, transferring to nitrocellulose, and probing with σ^32^. σ^32^ was detected at the position of full-length Ffh and the M-domain, but not at the position of the NG-domain (Figure 2D), indicating that the M-domain contains the determinants mediating the σ^32^-interaction.

**Figure 3:**
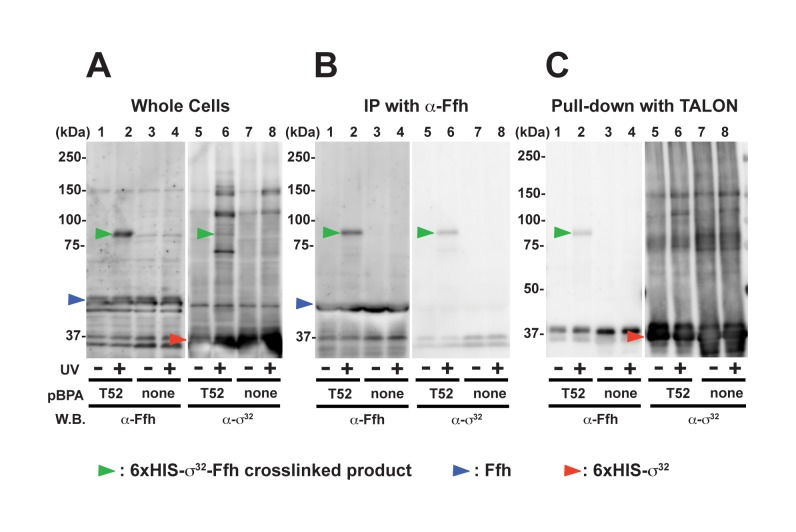
*In vivo* cross-linking between σ^32^ and Ffh. (A) Detection of a cross-linked product following UV irradiation in whole cells. Cells of CAG48238/pEVOL-pBpF/p6XH*-rpoHT52amber* (lanes 1, 2, 5, and 6) and CAG48238/pEVOL-pBpF/p6XH*-rpoH* (lanes 3, 4, 7, and 8) were grown at 30°C in L-medium supplemented with 0.02% arabinose, induced with 1 mM IPTG for 1 h, and UV-irradiated for 0 or 10 min as indicated. Total cellular proteins were acid-precipitated and analyzed by SDS-PAGE and immunoblotting with anti-Ffh and anti-σ^32^ antibodies. (B) Immunoprecipitation with anti-Ffh reveals a unique cross-linked product that interacts with anti-σ^32^. Supernatants of sonically disrupted UV-irradiated cells were subjected to immunoprecipitation with anti-Ffh antibodies. Immunocomplexes were solubilized in SDS sample buffer, analyzed by SDS-PAGE, and immunoblotted with anti-Ffh and anti-σ^32^ antibodies. Proteins from approximately 4.4-fold more cells were loaded onto the gel for the immunoprecipitated samples as compared with the whole cell samples. (C) Purification of 6×H-σ^32^ from UV-irradiated cells reveals a band that interacts with anti-Ffh. Supernatants of sonically disrupted UV-irradiated cells were subjected to TALON affinity chromatography, and bound proteins were eluted with 300 mM imidazole. Proteins in the eluate were acid-precipitated and analyzed by SDS-PAGE and immunoblotting with anti-Ffh antibodies. Proteins form approximately 20-fold more cells were loaded onto the gel for the TALON-affinity isolated samples as compared with the whole cell samples.

We used *in vivo* crosslinking to validate the direct interaction of SRP (Ffh+4.5S RNA) and σ^32^. We created a σ^32^ derivative with an N-terminal 6×HIS-tag and a photoreactive amino acid analog (*p*BPA) at amino acid position 52 (6×HIS-σ^32^T52*p*BPA; see Methods), which is active as WTσ^32^ in expression of the σ^32^ reporter P*_htpG_*-*lacZ* (activity is 150% that of WT; within the range of the variability of the assay; unpublished data). Following UV irradiation of whole cells, anti-Ffh immunoblotting of the whole cell lysate detected one predominant crosslinked product, which was dependent on UV-irradiation (Figure 3A, lanes 1 and 2) and *p*BPA at position 52 (Figure 3A, lanes 2 and 4). This UV- and *p*BPA-dependent product was also detected with anti-σ^32^ immunoblotting (Figure 3A, lane 6). To determine whether the crosslinked product represented 6×HIS-σ^32^T52*p*BPA-Ffh, we determined whether this product was identified both by co-immunoprecipitation with anti-Ffh antisera (Figure 3B) and by affinity purification of 6×HIS-σ^32^T52*p*BPA on a TALON resin (Figure 3C). Upon immunoprecipitation with anti-Ffh antisera, we detected a single higher molecular mass band, which reacted with both anti-Ffh (Figure 3B, lane 2) and -σ^32^ (Figure 3B, lane 6). Upon affinity purification on a TALON resin, anti-Ffh identified the same predominant UV- and *p*BPA-dependent Ffh-containing crosslinked product (compare Figure 3B and 3C, lane 2). Importantly, no free Ffh was recovered following TALON purification, indicating that the recovery of the Ffh conjugate was mediated by the covalently linked 6×HIS-σ^32^, rather than interaction with either the TALON resin or another protein. These results strongly suggest that σ^32^ directly interacts with Ffh *in vivo*. Although only a faint band was seen at the same position using anti-σ^32^ immunoblotting, this was likely a result of high background in this area of the gel, possibly because of extensive interaction between chaperones and σ^32^ (Figure 3C, lanes 5–8).

### I54Nσ^32^ Is Defective in Interacting with SRP

**Figure 4:**
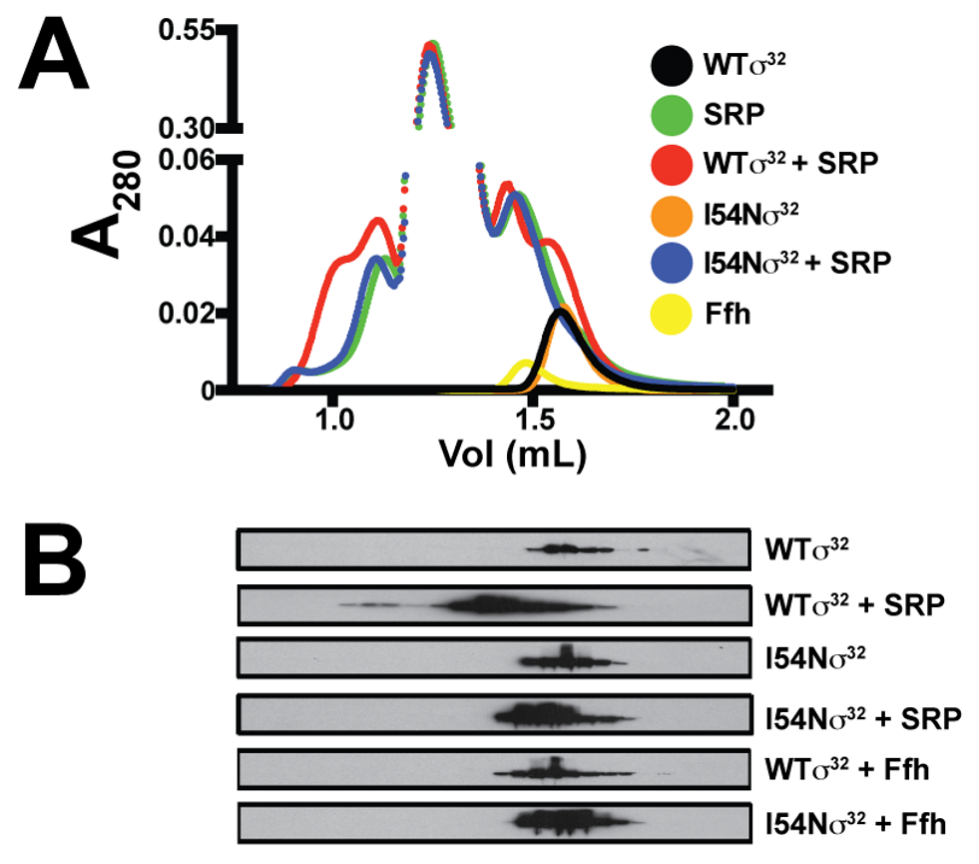
SRP (Ffh+4.5S RNA) preferentially interacts with WTσ^32^. (A) A_280_ elution profiles of WTσ^32^, I54Nσ^32^, Ffh, and SRP alone or in complex. WTσ^32^ or I54Nσ^32^ was incubated with a 10-fold molar excess of purified SRP on ice for 10 min, and complexes were analyzed by gel filtration on a Superdex 200 PC3.2/30 column. Protein elution was monitored by A_280_. Gel filtration of purified WTσ^32^, I54Nσ^32^, and SRP alone was carried out to determine the migration of each individual protein on the column. (B) Eluted fractions were separated on SDS-PAGE and probed with polyclonal antibodies against Ffh and σ^32^; Western blots of σ^32^ are shown. Experiments were performed at least four times, and a representative experiment is shown.

The function of the homeostatic control region of σ^32^ is not known [25]. I54Nσ^32^ is a mutation located in this region is severely compromised in both activity and degradation control, but the mechanism responsible for this phenotype had not yet been determined [25]. We therefore compared the binding of WTσ^32^ and I54Nσ^32^ to SRP using gel filtration. We incubated WTσ^32^ or I54Nσ^32^ either alone or in combination with SRP and subjected the mixture to gel filtration. Analysis of the elution profiles demonstrated that most WTσ^32^ was shifted towards the higher molecular weight region in the presence of SRP, and additionally, a fraction of σ^32^ eluted at a higher molecular weight than that of SRP alone, indicative of an SRP–σ^32^ complex [compare A_280_ profiles of σ^32^, SRP, and SRP-σ^32^ (Figure 4A) with immunoblotting for σ^32^ (Figure 4B; rows 1,2)]. σ^32^ present at a molecular weight between σ^32^ and SRP likely represents transient forms of the σ^32^–SRP complex. In sharp contrast, an interaction between I54Nσ^32^ with SRP was almost undetectable [compare A_280_ profiles of I54Nσ^32^ and SRP (Figure 4A) with immunoblotting for I54Nσ^32^ (Figure 4B; rows 3,4)], indicating that I54Nσ^32^ bound more weakly to SRP than WTσ^32^. Neither WTσ^32^ nor I54Nσ^32^ interacted detectably with Ffh, indicating that differential binding is dependent on the formation of SRP (Ffh+4.5S RNA), the biologically relevant cellular species of Ffh.

### σ^32^ Is Partially Membrane Associated in an SRP-Dependent Process

**Figure 5:**
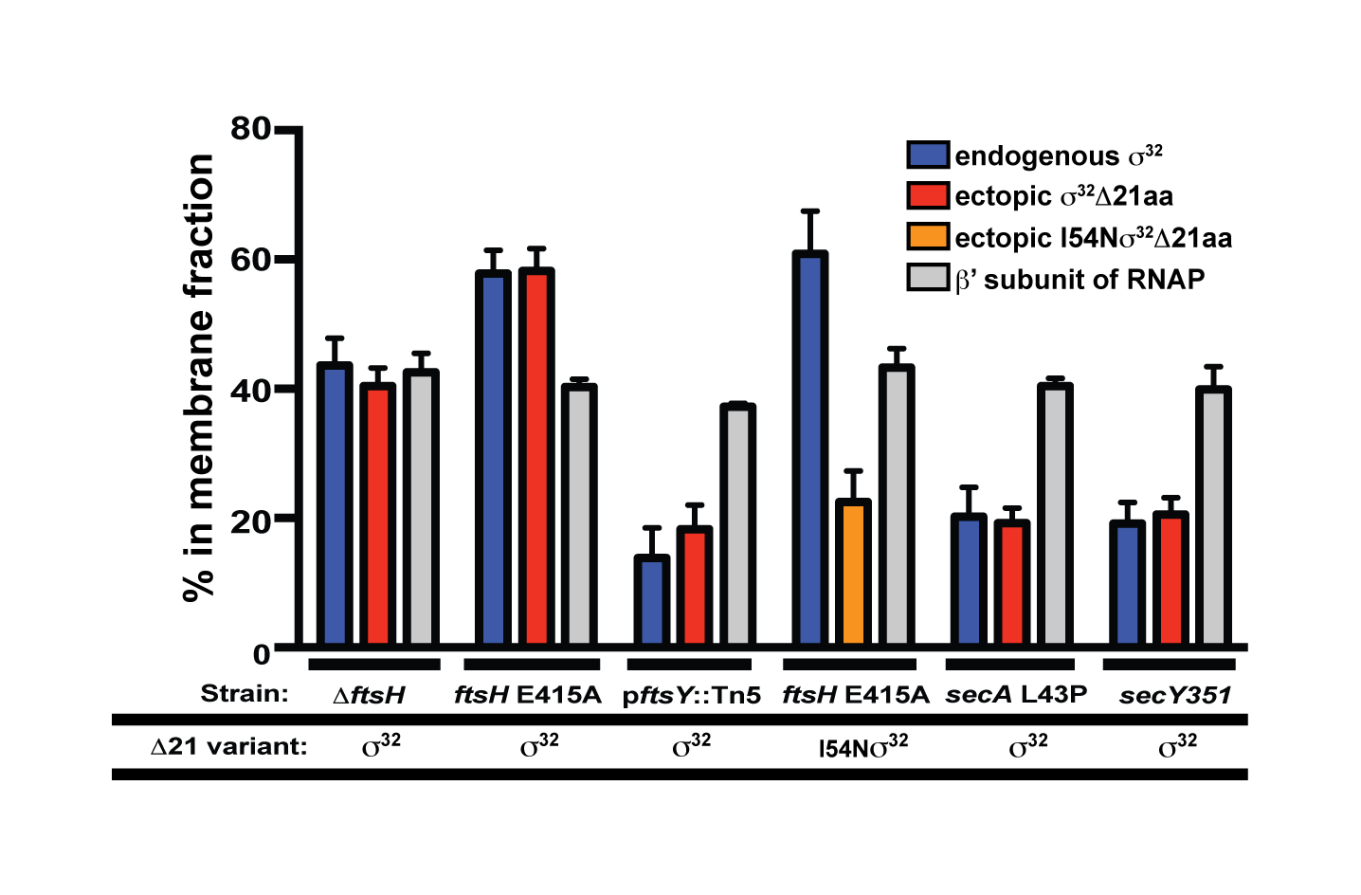
σ^32^ is partially membrane associated. The extent of association of σ^32^ and the β′ subunit of RNA polymerase with the membrane fraction was determined by quantitative immunoblotting of the soluble and nonsoluble fractions. Membrane association of σ^32^ and β′ was assessed in several relevant strain backgrounds. In addition to endogenous σ^32^, all strains contained a plasmid-encoded variant of σ^32^ lacking its 21 C-terminal amino acids (σ^32^Δ21aa). Ectopically expressed σ^32^Δ21aa or I54Nσ^32^Δ21aa were present at levels comparable to native σ^32^ and were distinguished from endogenous σ^32^ on a 12% SDS-PAGE gel. All fractionation experiments were performed ≥8 times, and % fractionation was calculated from experiments where probed cytoplasmic (RuvB) and membrane (RseA) proteins separated properly.

The biological function of SRP is co-translational protein targeting, leading us to test whether σ^32^ may be targeted to the IM through an SRP-dependent mechanism. Rapid degradation by FtsH normally keeps σ^32^ levels very close to the detection limit (∼20–50 molecules/cell; [8]), making reproducible detection following fractionation very difficult. Therefore, we performed fractionation experiments (Figure 5), either in cells expressing an enzymatically inactive mutant of the FtsH protease (FtsH E415A) or in cells lacking FtsH altogether (Δ*ftsH*). Approximately 44% of σ^32^ fractionated to the membrane in a Δ*ftsH* strain, and this fraction was increased to ∼58% in the FtsH E415A strain, raising the possibility that FtsH itself may participate in retention of σ^32^ at the IM. As the β′ subunit of RNA polymerase, a known interaction partner of σ^32^, also fractionated with the membrane, we next tested whether σ^32^ association with the IM was dependent on its association with RNA polymerase. To this end, we used σ^32^Δ21aa, which is defective in interacting with RNA polymerase [32]. We confirmed that σ^32^Δ21aa did not detectably interact with RNA polymerase (Figure S1A,B). Yet endogenous WTσ^32^ and ectopically expressed σ^32^Δ21aa fractionated equivalently to the IM both in Δ*ftsH* cells (∼39%) and in FtsH E415A cells (∼58%) (Figure S2), indicating that σ^32^ transited to the membrane independent of RNA polymerase.

We next tested whether the p*ftsY*::Tn*5* mutation or the homeostatic control region mutation of σ^32^ disrupted membrane partitioning of σ^32^. Both WTσ^32^ and ectopically expressed σ^32^Δ21aa were defective in partitioning to the IM in p*ftsY*::Tn*5* cells (Figure 5). To look at the effect of disrupting the homeostatic control region on membrane fractionation, we expressed I54Nσ^32^ as a σ^32^Δ21aa variant (I54Nσ^32^Δ21aa). The size difference allowed us to compare I54Nσ^32^Δ21aa and WTσ^32^ in the same cells (Figure S2). Whereas WTσ^32^ exhibited normal fractionation, I54Nσ^32^Δ21aa showed a severe localization defect, comparable to that of p*ftsY*::Tn*5* cells (Figure 5). We conclude that σ^32^ targeting to the IM is dependent on both SRP/SR and the σ^32^ homeostatic control region.

### Both SecA and SecY Are Important for Membrane Association of σ^32^

SecA is an ATP-fueled motor protein that recognizes signal peptides, drives the translocation of secreted proteins through the Sec translocon [33]–[37], and collaborates with the SRP/SR for integration of a subset of IM proteins into the membrane [33],[38]. We previously found that σ^32^ activity is increased in a SecA(*ts*) strain [39]. This observation motivated us to explore the relationship of SecA to IM trafficking of σ^32^. Indeed, using a SecA(*ts*) mutant with general defects in protein export (SecAL43P) [40],[41], we observed that cells displayed a significant defect in membrane localization of σ^32^ (Figure 5), as well as increased σ^32^ activity ([39] and unpublished data). In addition, purified SecA, resolved on SDS-PAGE and transferred to nitrocellulose, showed binding affinity for σ^32^, suggesting that these two proteins interact (Figure S3). We conclude that SecA participates in trafficking of σ^32^ to the IM.

**Figure 6:**
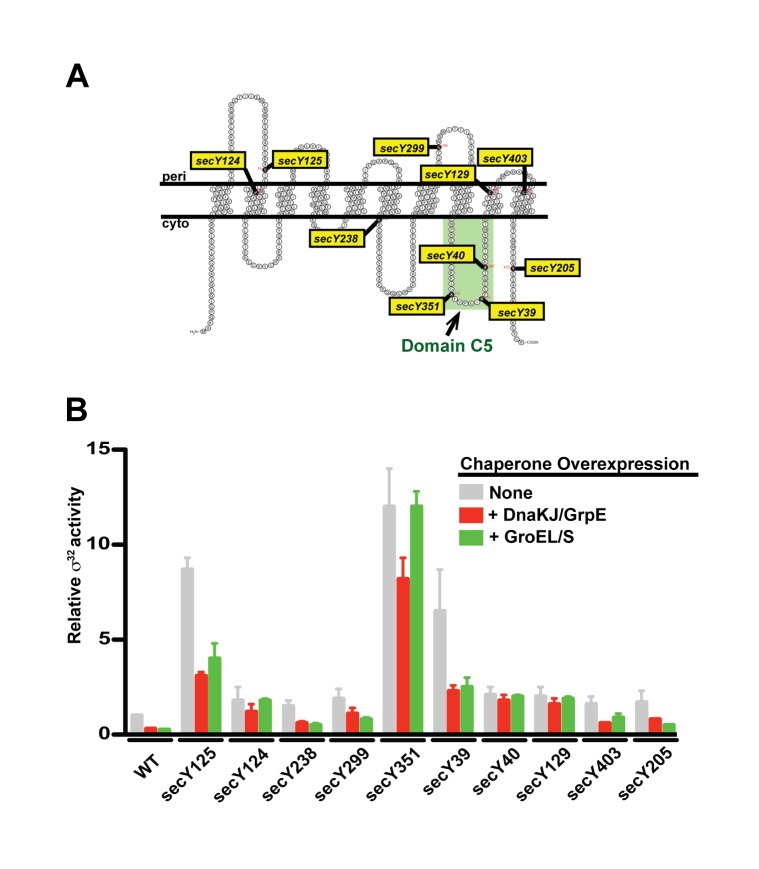
The SecY translocon plays a role in chaperone-mediated activity control of σ^32^. (A) Schematic of SecY topology in the IM by highlighting in yellow the locations/allele names of the mutated residues used in this study [51]. The region that interacts with FtsY (Domain C5) is boxed in green. (B) Mutations in *secY* show higher σ^32^ activity and affect chaperone-mediated activity control of σ^32^. The activity of σ^32^ was measured in WT and *secY* mutant cells growing at 30°C in LB medium (column 1) or in the same cells following induction of DnaK/J (column 2) or GroEL/S (column 3). Activity is calculated as the differential rate of β-galactosidase synthesis from a chromosomal P*_tpG_*-*lacZ* reporter in each cell type relative to that of WT cells.

SecY forms the core of the SecYEG IM translocon. This multidomain protein has a large cytoplasmic domain (C5) that functionally interacts with SR [42], SecA, and the ribosome [43]–[50] (Figure 6A). We tested whether 10 previously described *secY* mutations located in various domains of SecY (Figure 6A) [51] perturb chaperone-mediated control of σ^32^ activity and trafficking of σ^32^ to the IM (Figure 6B). All mutants had enhanced σ^32^ activity. This result was not surprising as *secY* mutants are expected to accumulate secretory protein precursors that titrate chaperones [52]. Importantly, four mutants (*secY124*, *secY351*, *secY40*, *secY129*) were also defective in chaperone-mediated control of σ^32^ activity (Figure 6B), as indicated by a lack of down-regulation of σ^32^ activity in response to overexpression of one or both of the chaperone systems. We examined the *secY351* mutant, which had both high σ^32^ activity and a significant defect in chaperone-mediated inactivation, and found it to be defective in IM trafficking of σ^32^ (Figure 5). *secY40* and *secY351* affect domain C5 (Figure 6A), implicated in the interaction of SecY with SR, raising the possibility that this interaction is important for both homeostatic control and IM targeting of σ^32^.

### An Independent Methodology Indicates Association of σ^32^ with the IM

Alkaline phosphatase is active only in the periplasm, where it forms the disulfide bonds necessary for its activity. Therefore, translational fusions to alkaline phosphatase (PhoA) lacking its own export signal are commonly used as an indicator of membrane targeting by the appended N-terminal sequence [53]. If the appended N-terminal sequence has either an export or insertion sequence, the fusion protein will exhibit alkaline phosphatase activity *in vivo* because it is partly transported to the periplasmic side of the membrane through the SecYEG translocon. Although σ^32^ has neither a membrane insertion nor an export sequence, it may contain a sequence that targets it to the cytosolic face of the IM. There is some evidence that the secretory apparatus can recognize the mature domains of exported proteins at low efficiency [54]. If so, proximity of PhoA to the translocon resulting from the IM targeting signal might enable transit of some fraction of PhoA to localize to the periplasmic side of the membrane, where it is active. By random insertion of the transposon probe Tn*phoA* into *rpoH*, encoding σ^32^ (see Materials and Methods), we found that a *phoA* fusion to the first 52 amino acids of σ^32^ (N52-σ^32^-PhoA) showed ∼10-fold greater PhoA activity than signal-less PhoA itself, indicating that the N-terminus of σ^32^ facilitates PhoA export (Table 2). Moreover, PhoA activity enhancement is dependent both on the SRP/SR-dependent trafficking system and on SecY, as both p*ftsY*::Tn*5* and *secY351* decreased the PhoA activity ∼2-fold, whereas leaderless PhoA exhibited little response to these perturbations (Table 2). Thus, this assay is consistent with the idea that the N-terminus of σ^32^ carries an IM-trafficking sequence and that the targeting process is dependent on SRP and SecY.

**Table 2:** N-terminal segment of σ^32^ directs activation of PhoA protein through the SRP-Sec pathway.

	N52-σ^32^-PhoA		Signal-Less PhoA	
Strain	PhoA Activity	Protein Level	PhoA Activity	Protein Level
Wild-type	1.00±0.11*	1.0	0.11±0.03	0.15
p*ftsY*::Tn5	0.43±0.05	0.8	0.14±0.03	0.2
*secY351*	0.46±0.07	1.5	0.32±0.05	0.2

Wild-type strain (MG1655) and its derivatives carrying the mutation as indicated were transformed by the plasmid containing each PhoA construct. The resulting transformants were grown to log phase in LB medium at 30°C. PhoA activity and protein levels were determined by standard procedures (see Materials and Methods).

* The activity of N52-σ^32^-PhoA was set to 1.00 in the wild-type strain.

### Membrane-Tethering of Otherwise Deregulated σ^32^ Restores Homeostatic Control

The I54Nσ^32^ mutant and mutants in the IM-targeting machinery (p*ftsY*::Tn*5*, *secA*(*ts*), *secY351*) were both defective in proper regulation of σ^32^ and in σ^32^ association with the IM. This convergence motivated us to test whether artificially tethering σ^32^ to the IM could restore homeostatic control. To this end, we exploited the bacteriophage Pf3 coat protein. With the addition of three leucine residues in its membrane-spanning region, 3L-Pf3 translocates spontaneously in an orientation-specific manner to the IM, where it inserts in an N-out/C-in orientation [55]. We modified *rpoH* (encoding σ^32^) at its chromosomal locus to encode a σ^32^ variant with the 3L-Pf3 membrane-insertion signal attached to its N-terminus (schematized in Figure S4A). Strains carrying 3L-Pf3-σ^32^ (IM-WTσ^32^) or 3L-Pf3-I54Nσ^32^ (IM-I54Nσ^32^) as their sole source of σ^32^ were viable, even though 99% of IM-WTσ^32^ was inserted in the membrane as judged by fractionation studies (Figure S4B). Thus, σ^32^ functions when it is tethered to the IM.

We determined whether IM-WTσ^32^ was subject to homeostatic control circuitry exhibited by WTσ^32^. σ^32^ is maintained at a low level by FtsH degradation, and its activity is decreased by chaperone-mediated inactivation. Both phenotypes are evident by comparing the amount and activity of σ^32^ in a WT versus a Δ*ftsH* strain. In a Δ*ftsH* strain, the level of WTσ^32^ increases ∼30-fold because the major protease degrading σ^32^ is removed (Table 3; Figure S5 [compare lanes 1 and 3]; and [25]). However, the activity of σ^32^ increases only 3-fold as a consequence of chaperone-mediated activity control, leading to a 10-fold reduction in the S.A. of σ^32^ in Δ*ftsH* cells relative to that in WT cells (Table 3 and [56]). Both the level and S.A. of WTσ^32^ and IM-WTσ^32^ were closely similar in a Δ*ftsH* strain, indicating that the chaperone-mediated activity control circuit is active in IM-WTσ^32^ (Table 3 and Figure S5 [compare lanes 3 and 4]). Additionally, the level of IM-WTσ^32^ was significantly lower in *ftsH* ^+^ than in a Δ*ftsH* strain, indicating that IM-WTσ^32^ was efficiently degraded by FtsH (Table 3 and Figure S5 [compare lanes 2 and 4]). The presence of a contaminating band prevented absolute quantification of IM-WTσ^32^ levels via Western blot analysis (Figure S5). However, if the relative S.A. of IM-WTσ^32^ and WTσ^32^ are equivalent in the *ftsH* ^+^ strain as we found in the Δ*ftsH* strain, then the 2-fold decrease in activity of IM-WTσ^32^ relative to WTσ^32^ implies a slight increase in the rate of degradation of IM-WTσ^32^ relative to WTσ^32^. Note that the 3L-Pf3 membrane-insertion tag itself is not a signal for FtsH degradation, as the stability of the FliA σ factor, which is closely related to σ^32^, was unchanged when expressed as 3L-Pf3-FliA (Figure S6). In summary, both the chaperone-mediated activity control circuit and the FtsH-mediated degradation control circuit are active on IM-tethered σ^32^.

**Table 3:** IM-insertion of σ^32^ significantly restores homeostatic control to mutant cells.

	σ^32^ Level		σ^32^ Activity		Relative S.A. of σ^32^	
σ^32^ Variant	WT	Δ*ftsH*	WT	Δ*ftsH*	WT	Δ*ftsH*
σ^32^	1.0±0.1	32.1±5.0	1.0±0.1	3.1±0.1	1.0	0.1
IM-σ^32^	a	26.7±4.5	0.7±0.1	2.0±0.1	N.D.	0.1
I54Nσ^32^	11.4±2.1	b	6.7±0.5	b	0.6	N.D.
IM-I54Nσ^32^	a	29.5±6.0	1.3±0.6	4.3±0.8	N.D.	0.1
p*ftsY*::Tn5	6.8±0.6		7.7±1.0		1.1	
p*ftsY*::Tn5; IM-σ^32^	a		0.7±0.1		N.D.	

Protein levels were determined by SDS-PAGE followed by immunoblotting, and averages from 3–4 expts are presented. Relative S.A., relative Specific Activity, is calculated as described in Table 1. N.D., not determined, denotes that values could not be determined because of ^a^ and ^b^.

a Levels of the σ^32^ variants could not be measured accurately.

b An I54Nσ^32^Δ*ftsH* strain is inviable.

Next, we asked whether the forced and stable tethering of σ^32^ to the IM bypassed the regulatory defects of I54Nσ^32^ and the reduced-level SR mutant p*ftsY*:::Tn*5*. I54Nσ^32^ is degraded poorly by FtsH as its level was 11-fold higher than that of WTσ^32^ (Table 3; Figure S5 [compare lanes 1 and 6] and [25]). I54Nσ^32^ also had compromised chaperone-mediated activity control as the high chaperone levels in this strain did not reduce the S.A. of I54Nσ^32^ (Table 3; and [25]). In stark contrast, both degradation and activity control were restored when I54Nσ^32^ was converted to IM-I54Nσ^32^. FtsH efficiently degraded the membrane-tethered variant: IM-I54Nσ^32^ was undetectable in *ftsH* ^+^ cells but present at a high level in Δ*ftsH* cells (Table 3 and Figure S5 [compare lanes 5 and 7]). Additionally, IM-I54Nσ^32^ and IM-WTσ^32^ exhibited comparable reductions in relative S.A. of σ^32^ in Δ*ftsH* cells (Table 3). Stable tethering of σ^32^ to the IM also bypassed the regulatory defects of p*ftsY*::Tn*5* as IM-WTσ^32^ in the reduced-level SR background was degraded and subject to chaperone-mediated activity control. Indeed, IM-WTσ^32^ behaved identically in WT and p*ftsY*::Tn*5* strains, exhibiting comparable σ^32^ activity at a protein level below detection (Table 3 and Figure S5 [compare lanes 8 and 9]). Finally, IM-tethering relieved the growth defects of both I54Nσ^32^ (Figure S7A and C) and of p*ftsY*::Tn*5* (Figure S7B, C, and D). In summary, stable tethering of σ^32^ to the IM restored both homeostatic control and normal growth to cells with a defective σ^32^ homeostatic control region and to cells with a compromised SRP/SR co-translational targeting apparatus.

## Discussion

Our work has led to a revised model of the HSR circuitry (Figure 1B). σ^32^ first transits to the IM via an SRP/SR-dependent process and is then subjected to the chaperone-mediated activity control and FtsH-mediated degradation control that have been previously described. This revised model enables the homeostatic control circuit to integrate information on both cytosolic and IM status. Importantly, the efficiency of co-translational protein targeting depends on the cumulative effect of multiple SRP checkpoints including differences in cargo binding affinities, kinetics of SRP-SR complex assembly, and GTP hydrolysis [57]. Multiple checkpoints and the fact that SRP is sub-stoichiometric relative to translating ribosomes (∼1∶100; SRP molecules to translating ribosomes [58]) may allow SRP to modulate the extent of IM-localization of σ^32^ during times of stress and/or increased protein flux. Thus, σ^32^ down-regulation through its localization to the membrane could be alleviated when the IM is disturbed or SRP is overloaded in assisting membrane protein biogenesis. This feed-forward mechanism allows the σ^32^ homeostatic control to sense the state of cytosolic and IM proteostasis before unfolded proteins accumulate to a significant extent. Interestingly, *ffh* (encoding the protein subunit of the SRP) is a σ^32^ regulon member as its expression increases at least 3-fold following induction of σ^32^ either by heat shock or by deletion of *dnaK/J* ([30] and unpublished data). This could provide an additional connection between σ^32^ and protein flux to the IM. Finally, and more speculatively, given the demonstrated involvement of SecA in IM targeting of σ^32^ and its direct interaction with σ^32^, the σ^32^ homeostatic control circuit may also monitor protein flux through SecA to the periplasm and outer membrane.

The idea that the high activity of σ^32^ in the I54Nσ^32^ homeostatic control mutant and in SRP/SR mutants (eg. p*ftsY*::Tn*5*) results from σ^32^ mislocalization to the cytosol and consequent homeostatic dysregulation, rather than from chaperone titration by a buildup of unfolded proteins, is supported by our data. First, forced IM-tethering overcomes the inviability of the I54Nσ^32^ mutation in the *ΔftsH* strain background (Table 3), as well as the growth defects of I54Nσ^32^ and p*ftsY*::Tn*5* (Figure S7), suggesting that high expression of σ^32^ is aberrant and deleterious to cells, rather than required to remodel misfolded proteins. This is reminiscent of previous findings that reduced-function σ^32^ mutants suppress physiological defects of a Δ*dnaK* strain [59] and that overexpression of HSPs was deleterious to growth [13],[60]. Second, *secY* mutants dysregulated in chaperone-mediated activity control were not distinguished by their extent of σ^32^ induction. This is contrary to the prediction of the chaperone titration model, which posits that *secY* mutants with the highest σ^32^ induction would have the highest level of unfolded proteins. These mutants would then be refractory to activity control because the additional chaperones resulting from chaperone overexpression would actually be needed to remodel the misfolded protein burden. We conclude that homeostatic dysregulation of σ^32^ results from σ^32^ mislocalization, rather than from the buildup of unfolded proteins.

The molecular nature of IM-localized σ^32^ remains unclear. Prediction programs [61],[62] do not detect either a signal peptide-like or transmembrane sequence in σ^32^. We favor the idea that following transit to the IM, σ^32^ is maintained at the membrane via interactions with other proteins and/or lipid head groups during its short half-life in the cell (30–60″). Indeed, we have already demonstrated interactions between σ^32^ and several membrane-associated or IM proteins, including SRP, SecA, and FtsH itself. Moreover, the chaperone systems regulating σ^32^ (DnaK/J/GrpE and GroEL/S) show partial distribution to the membrane [63]–[68], whereas other potential membrane-associated protein partners have not yet been tested for σ^32^ interaction (e.g., SecY and additional members of the Sec machinery). Each of these proteins could result in partial membrane localization of σ^32^, as was shown for FtsH where deletion of the protein decreased localization relative to cells with the protease-dead mutation FtsH E415A. Importantly, if σ^32^ is membrane associated via transient protein–protein and/or protein–lipid interactions, some σ^32^ may dissociate from the membrane during cell lysis, as was demonstrated for FtsY, another peripheral membrane protein [69],[70]. Therefore, although we report that ∼50% of σ^32^ is membrane-associated, the fraction of σ^32^ that is actually IM-localized may be significantly higher.

IM-associated σ^32^ may provide regulatory flexibility not possible for IM-tethered σ^32^. For example, during times of high stress, σ^32^ may be able to dissociate from the membrane to escape homeostatic control. These excursions could be transient if SRP were able to transport σ^32^ posttranslationally, a possibility suggested by the fact that full-length, fully folded σ^32^ binds to SRP (Figures 2 and 3 and Figure S1). Additionally, IM-tethered σ^32^ is more rapidly degraded than IM-associated σ^32^, suggesting that tethering makes σ^32^ a better FtsH substrate. This could diminish the ability of the cell to regulate the rate at which FtsH degrades σ^32^, which is of physiological significance during temperature upshift [8]. The transient reduction in σ^32^ degradation following increased temperature contributes significantly to the rapid build-up of σ^32^ during heat shock [8].

Membrane localization is widely used to control σ factors [71],[72]. The inactive *B. subtilis* SigK pro-protein is membrane inserted; cleavage of its N-terminal pro-sequence releases SigK [73],[74]. Cleavage is coordinated with passage of a checkpoint in spore development to provide just-in-time SigK activity [75]. Additionally, many σ factors are held in an inactive state at the membrane by cognate membrane-spanning anti-σ factors and released as transcriptionally active proteins when stress signals lead to degradation of their anti-σ [71],[76]. IM-localization of σ^32^ serves a conceptually distinct role as σ^32^ is equally active in the cytoplasm or at the IM. Instead, the localization process itself is the key regulatory step in two ways: localization is both regulated by protein folding status and is prerequisite for proper function of the homeostatic control circuit.

The SRP-SR co-translational targeting system has an important role in maintaining proteostasis. SRP-SR minimizes aggregation and misfolding of the approximately 20%–30% of proteins destined for the IM, by making their translation coincident with membrane insertion. Our finding, that SRP/SR-mediated transit of σ^32^ to the IM is also critical for proper control of the HSR, points to a significant new regulatory role for the co-translational targeting apparatus in protein-folding homeostasis. This finding also raises important mechanistic questions. Our *in vitro* interaction results suggest a direct, but weak, interaction between full-length σ^32^ and the M-domain of SRP. The prevailing paradigm suggests that the M-domain interacts only with nascent polypeptides with particularly hydrophobic signal sequences. It is possible that σ^32^ is detected co-translationally, as the Region 2.1 N-terminal α-helical structure, which resembles a hydrophobic signal sequence, may be recognized by the SRP. Alternatively, we note that the SRP chloroplast homolog (cpSRP54) has a dedicated posttranslational targeting mechanism for several fully translated membrane proteins [77], and *E. coli* SRP, alone or in combination with additional accessory factors (e.g., other σ^32^ interactors, such as chaperones or SecA), may target mature σ^32^ to the membrane *in vivo*. It remains to be determined whether an interaction between full-length σ^32^ and SRP, or a novel co-translational targeting interaction by the SRP-SR system, mediates transit of σ^32^ to the membrane.

## Materials and Methods

### Strains, Plasmids, and Growth Conditions

All strains used were derivatives of the *E. coli* K-12 strain MG1655, CAG48238 [25],[39]. For chaperone overexpression experiments, mutations were transduced with phage P1 into strains carrying chromosomal P*_ara_*-*groEL/S* [78] or P_A1/lacO-1_-*dnaK/J-lacI^q^* [14]. Mutant alleles in *secY* [51] and *secA* [39] were transferred to various strain backgrounds through P1 transduction. The SecAL43P mutant used here is a SecA(*ts*) allele, with general defects in protein export [40],[41]. For propagation and transfer of the R6K *pir* plasmid, pKNG101, strains DH5σ λ*pir* and SM10 λ*pir* were used, respectively. Plasmids pET21a and pTrc99A were used as expression plasmids. For construction of pRM5 (6×HIS-*rpoH*), the *rpoH* gene was PCR-amplified from the chromosomal DNA of W3110 and cloned into the EcoRI-SalI sites of pTTQ18 [79]. Then, the T52amber mutation was introduced into pRM5 by site-directed mutagenesis, yielding pRM17 (6×HIS-σ^32^T52amber). pEVOL-pBpF (Addgene) carried evolved *Methanocaldococcus jannaschii* aminoacyl-tRNA synthetase/suppressor tRNA for incorporation of a photoreactive amino acid analog, *p*-benzoylphenylalanine (*p*BPA), into the amber codon site. All strains were grown in LB medium. When required, antibiotics were added to the medium as follows: 100 µg/ml ampicillin, 30 µg/ml kanamycin, 20 µg/ml chloramphenicol, and 25 µg/mL streptomycin.

### Isolation of p*ftsY*::Tn*5* Mutant

Strain CAG48275 [25], which is *ΔlacX74*, contains the prophage JW2 (P*_htpG_*-*lacZ*), and a chromosomal *dnaK/J* locus driven from P_A1/lacO-1_ under control of *lacI^q^* [14] was grown in LB, induced with 1 mM IPTG to overexpress DnaK/J chaperones, treated with Tn*5*, and plated at 30°C on X-gal indicator plates containing kanamycin to select for strains containing Tn*5*. Blue colonies were picked and tested for higher σ**^32^** activity and for feedback resistance to excess DnaK/J [25]. Tn*5* insertion sites were determined by DNA sequencing.

### β-Galactosidase Assay

Overnight cultures (LB medium) were diluted 250-fold and grown to exponential phase (OD_600_ = 0.05–0.5). Samples were taken at intervals starting at OD_600_ = 0.05, and σ^32^ activity was monitored by measuring β-galactosidase activity expressed from the σ^32^-dependent *htpG* promoter, as done previously [25].

### Protein Purification

The following proteins were purified essentially as described: 6×H-tagged, Strep-6×H-tagged, and untagged WTσ^32^ or I54Nσ^32^ [80], FtsY, Ffh, 4.5S RNA [81], and SecA [82]. Chaperones were removed from σ^32^ with an additional wash containing 10 mM ATP, 10 mM MgCl_2_, and 25 uM of both peptides, CALLLSAARR and MQERITLKDYAM, synthesized by Elim Biopharmaceuticals, Inc (Hayward, CA).

### *In Vivo* Co-Immunoprecipitations

Cells were grown to OD_600_∼0.35 in LB medium at 30°C, harvested, washed two times with 1× PBS, resuspended in Lysis Buffer (20 mM Hepes-KOH, 150 mM NaCl, 10 mM EDTA, 10% glycerol, pH 7.5), and lysed by passing 4× through an Avestin EmulsiFlex-C5 cell homogenizer at 15,000 psi. Cellular debris was spun out and the supernatants were incubated with anti-Ffh or anti-FtsY antibodies at 4°C for 14 h by rotation. TrueBlot anti-Rabbit Ig IP Beads (eBioscience) were added and the supernatants rotated for an additional 2 h at 4°C. Immunocomplexes were isolated by centrifugation and washed 5× in Lysis Buffer without EDTA, and eluted in TCA Resuspension Buffer (100 mM Tris (pH 11.0), 3% SDS) containing LDS Sample Buffer (Invitrogen). Proteins were separated by 10% SDS-PAGE, analyzed by immunoblotting using anti-σ^70^ and anti-σ^32^ antibodies, and imaged using fluorescent secondary antibodies (as described below).

### Identification of Direct Protein–Protein/Domain Interactions

Detection of a direct protein–protein/domain interaction was carried out exactly as previously described [83]. Proteins were separated on 10% SDS-PAGE. Partially proteolyzed Ffh was obtained by incubating 400 µg of purified Ffh with 4 µg of Glu-C endopeptidase (New England Biolabs) at 25°C in 10 mM Na-HEPES (pH 7.5), 150 mM NaCl, 1 mM DTT, 10 mM MgCl_2_, and 10% glycerol. An aliquot of the reaction was taken out at various times (0, 5, 10, 15, 30, 45, 60, 120, 180, and 330 min) and stopped by addition of 5× volume of 5× SDS-sample loading buffer. The samples were then analyzed by blot overlay with σ^32^ as the probe.

### *In Vivo* Crosslinking, 6×HIS-tag Affinity Isolation and Co-Immunoprecipitation

*In vivo* crosslinking experiments were carried out essentially as described previously [84]. Strains of CAG48238 carrying pEVOL-pBpF were further transformed with pRM5 or pRM17. Cells were grown at 30°C in L medium containing 0.02% arabinose and 1 mM *p*BPA, induced with 1 mM IPTG for 1 h, and UV-irradiated for 0 or 10 min at 4°C. For analysis of whole cell samples, total cellular proteins were precipitated with 5% trichloroacetic acid, solublized in SDS sample buffer, and analyzed by 7.5% SDS-PAGE and immunoblotting.

Co-immunoprecipitations were carried out as follows: UV-irradiated cells were suspended in 10 mM Tris-HCl (pH 8.1) and disrupted by sonication at 0°C. After removal of total membranes by ultracentrifugation, proteins were precipitated with 5% trichloroacetic acid, washed with acetone, and solubilized in buffer containing 50 mM TrisHCl (pH 8.1), 1% SDS, 1 mM EDTA. The samples were then diluted 33-fold with NP40 buffer (50 mM TrisHCl (pH 8.1), 150 mM NaCl, 1% NP40). After clarification, supernatants were incubated with anti-Ffh antibodies and TrueBlot anti-Rabbit Ig IP Beads (eBioscience) at 4°C for 13 h with rotation. Immunocomplexes were isolated by centrifugation, washed 2 times with NP40 buffer and then once with 10 mM TrisHCl (pH 8.1), and dissolved in SDS sample buffer. Proteins were separated by 7.5% SDS-PAGE and analyzed by immunoblotting using anti-Ffh and anti-σ^32^ antibodies, TrueBlot anti-Rabbit IgG (eBioscience), and Can Get Signal immunoreaction enhancer solution (TOYOBO Life Science, Japan).

For 6×HIS-tag affinity isolation, UV-irradiated cells were suspended in 10 mM Tris-HCl (pH 8.1) containing 6 M urea and disrupted by sonication at 0°C. After clarification by ultracentrifugation, the soluble fraction was loaded onto the TALON resin (TAKARA BIO, Inc., Japan). After washing the resin with wash buffer (50 mM TrisHCl (pH 7.0), 300 mM KCl, 6 M urea, 20 mM imidazole), bound proteins were eluted with wash buffer containing 300 mM imidazole. Proteins were precipitated with 5% trichloroacetic acid, solublized in SDS sample buffer, and analyzed by 7.5% SDS-PAGE and immunoblotting.

### Gel Filtration

Purified proteins were run on a Superdex 200 PC 3.2/30 column, pre-equilibrated with Buffer A (20 mM Tris-HCl pH 8.0, 150 mM NaCl, 10 mM MgCl_2_, 2 mM DTT). Purified proteins or protein complexes were run with Buffer A at a flow rate of 40 µL/min, and collected fractions were analyzed by SDS-PAGE and immunoblotting for σ^32^. SRP was formed by incubating purified Ffh with 1.5× molar excess of purified 4.5S RNA on ice for 10 min. To form SRP-σ^32^ complexes, 3 µM of purified WTσ^32^ or I54Nσ^32^ was mixed with 10× molar excess of SRP; proteins were incubated on ice for 30 min before analysis by gel filtration.

### Construction of σ^32^-PhoA Fusion

A 52-σ^32^-Tn*5*PhoA fusion was initially isolated by random screening for PhoA^+^ clones on PhoA indicator plates—using a strain carrying a *TnphoA* transposon probe [85] on a low-copy plasmid and P*_lac_*-*rpoH* (encoding σ^32^) on a multicopy plasmid. The fusion used in this article (N52-σ^32^-PhoA lacking the transposon but containing the first 52 amino acids of WTσ^32^) was subsequently constructed by standard recombinant DNA techniques. Direct construction of fusions past amino acid 52 of σ^32^ was very unstable, precluding their analysis.

### Cell Fractionation

Cells were grown to OD_600_ = 0.3–0.4, harvested, and resuspended in ice-cold Buffer B (10 mM Tris-Acetate (pH 7.4), 10 mM Mg(OAc)_2_, 60 mM NH_4_Cl, 1 mM EDTA, supplemented with 1 mM PMSF) to an OD_600_ of 15. Cells were immediately lysed by passaging the extracts through an Avestin EmulsiFlex-C5 cell homogenizer at 15,000 psi, and subjected to low-speed centrifugation to remove cell debris and un-lysed cells. Membranes were collected by ultracentrifugation in an Optima benchtop centrifuge (Beckman–Spinco) with a TLA 100.3 rotor (60 min; 52,000 rpm; 4°C). The supernatant was saved as the soluble fraction, while the pellet was washed 3× with Buffer B and then resuspended in Buffer C (50 mM HEPES-KOH pH 7.6, 50 mM KCl, 1 mM EDTA, 1 mM EGTA, 0.5% *n*-Dodecyl β-D-maltoside, and 5% glycerol). Both the soluble and membrane fractions were precipitated in trichloroacetic acid (13% vol/vol), incubated on ice for 30 min, and then overnight at 4°C. Precipitated proteins were then washed with ice-cold acetone and analyzed by SDS-PAGE and immunoblotted for σ^32^ (Neoclone), β′ (Neoclone), σ^70^ (Neoclone), RseA [86], and RuvB (Abcam) with fluorescent secondary antibodies (LI-COR Biosciences) used for detection. The percentage of σ^32^ in each fraction was determined by direct scanning and analyzing bands with ImageJ software (National Institutes of Health).

### RNA Polymerase Pull-Downs

Cells were grown to OD_600_ = 0.35–0.45, harvested, and resuspended in ice-cold Buffer D (50 Tris-HCl, pH 8.0, 0.1 mM EDTA, 150 mM NaCl, and 5% glycerol) to an OD_600_ of 20. Lysozyme was added to 0.75 mg/mL and cells were incubated on ice for 30 min, followed by sonication, then subjected to low-speed centrifugation to remove cell debris and unlysed cells. Lysates were then incubated with pre-equilibrated, pre-blocked (Buffer D containing 5% Bovine Serum Albumin, 0.1 mg/mL dextran) Softag 4 Resin (Neoclone) overnight at 4°C. Bound proteins were washed 3× with Buffer D and eluted with 4× LDS NuPAGE Buffer (Life Technologies). To collect lysates and eluted proteins, 0.05 µM of Strep-6×H-tagged σ^32^ was added as a loading and blotting control during analysis by SDS-PAGE and Western blotting against σ^32^.

### Construction of 3L-Pf3 Fusion Proteins

The 3L-Pf3 genetic sequence was created by carrying out standard polymerase chain reaction using the following overlapping oligos: 5′-atgcaatccgtgattactgatgtgacaggccaactgacagcggtgcaagc-3′, 5′-taccattggtggtgctattcttctcctgattgttctggccgctgttgtgctggg-3′, 5′-aaagaattgcgctttgatccagcgaatacccagcacaacagcggccagaa-3′, and 5′-aagaatagcaccaccaatggtagtgatatcagcttgcaccgctgtcagtt-3′. The stitched oligos were then cloned using TOPO TA cloning (Invitrogen) and sequenced. To construct chromosomal 3L-Pf3-σ^32^, PCR was carried out to stitch the 3L-Pf3 gene sequence flanked by the first 500 base pairs of the σ^32^ open reading frame and 500 base pairs upstream of the start codon, and subsequently cloned into the pKNG101 suicide vector. The 3L-Pf3 sequence was then integrated 5′ and in-frame with the chromosomal *rpoH* gene by double homologous recombination. Counterselection of *sacB* on pKNG101 was carried out on 10% sucrose media (5 g/L Yeast Extract, 10 g/L Tryptone, 15 g/L Bacto Agar, 10% sucrose) [25],[87]. Clones were sequenced to verify chromosomal integration of the 3L-Pf3 sequence in the correct reading frame.

To construct pTrc99A expressing 3L-Pf3-FliA, *flgM* and *fliA* (in that order) were cloned as an operon, with the sequence 5′-ccgtctagaattaaagAGGAGaaaggtacc-3′ added between the two genes in the vector; the Shine-Dalgarno site is designated in uppercase. Two plasmids were created—one with just *flgM* and *fliA*, unmodified, and one where the 3L-Pf3 sequence was cloned 5′ to and in-frame with *fliA*. Clones were sequenced to verify correct sequences and proper reading frame. Expression was from the leaky pTrc promoter, and experiments were only carried out after fresh transformation into the parental CAG48238 strain. Levels of FliA were analyzed by SDS-PAGE and immunoblotting with antibodies against FliA (Abcam).

### Immunoblotting

Cells were re-suspended in equal volumes of Buffer C, with the addition of trichloroacetic acid (final 13% vol/vol), kept on ice overnight, and the precipitate collected by centrifugation. Pellets were washed with acetone and resuspended in 1× LDS NuPAGE Buffer (Life Technologies). Serial dilutions of WT and mutant samples were loaded onto a polyacrylamide gel, and proteins transferred to nitrocellulose membranes. The blots were first probed with primary antibodies and then with anti-primary fluorescence-conjugated secondary antibody (Licor). Immunoblots were scanned at the appropriate wavelengths for detection. Fold increase (protein level experiments) was estimated by comparison with a dilution series of samples from the WT strain. Fold decrease after addition of chloramphenicol (protein stability experiments) was determined by direct scanning and analyzing bands with ImageJ software (National Institutes of Health).

## Supporting Information

Figure S1 **σ^32^Δ21aa, a C-terminal truncation of σ^32^, is defective in binding to RNA polymerase** ***in vivo*** **.** (A) Immunoprecipitation of RNA polymerase-bound native σ^32^ and σ^32^Δ21aa. σ^32^Δ21aa was expressed from pTrc99A in Δ*ftsH* cells, induced to levels comparable to endogenous σ^32^, grown to mid-exponential at 30°C in LB medium and the amount of σ^32^ bound to the anti-β′ resin (Softag4; Neoclone) and remaining σ^32^ in the supernatant was quantified by immunoblotting using a polyclonal antibody against σ^32^. Comparable amounts of total cellular lysates (TCL; left lane) and corresponding RNA-polymerase immunoprecipitations (RNAP IP; right lane) are shown. Purified σ^32^, tagged at the N-terminus with a Strep and 6×Histidine (Strep-6×H) tag, was used as a loading and blotting control. Results of a representative experiment are shown. (B) Quantification of RNA polymerase-bound native σ^32^ and σ^32^Δ21aa expressed in the same strain background (Δ*ftsH*). Averages of four independent experiments are shown.
(TIF)

Figure S2 **Membrane fractionation of σ^32^ is independent of RNA polymerase binding.** *ftsH* E415A cells expressing either WTσ^32^Δ21aa or I54Nσ^32^Δ21aa were subjected to cellular fractionation (see Materials and Methods), and soluble and membrane fractions were resolved by SDS-PAGE and analyzed by immunoblotting for σ^32^, σ^70^, and the β′ subunit of RNA polymerase. Ectopically expressed σ^32^Δ21aa or I54Nσ^32^Δ21aa were present at levels comparable to native σ^32^ and were distinguished from endogenous σ^32^ on a 10% SDS-PAGE gel. All fractionation experiments were performed ≥8 times, and % fractionation was calculated from experiments where probed cytoplasmic (RuvB) and membrane (RseA) proteins separated properly.
(TIF)

Figure S3 **σ^32^ interacts with SecA through protein–protein interaction analysis.** Purified SecA was run on a 10% SDS-PAGE gel (along with FtsY and Ffh), transferred to nitrocellulose, re-natured, and incubated with purified WTσ^32^. The Coomassie-stained gel of the prey proteins (FtsY, Ffh, and SecA; left) and the nitrocellulose membrane containing the transferred prey proteins, probed with polyclonal anti-σ^32^ antibodies (right), are shown. The Coomassie-stained gel section of FtsY and Ffh and the corresponding σ^32^-incubated nitrocellulose membrane probed with anti-σ^32^ antibodies are also shown in Figure 2C.
(TIF)

Figure S4 **Fusing the 3L-Pf3 peptide to the N-terminus of WTσ^32^ coding sequence significantly increases its membrane localization.** (A) Schematic representation of membrane-tethered 3L-Pf3-WTσ^32^ (IM-WTσ^32^). The amino acids corresponding to the 3L-Pf3 and σ^32^ are shown as open or enclosed dark circles, respectively. (B) Soluble (lanes 1 and 3) and membrane (lanes 2 and 4) fractions from cellular fractionations (described in Materials and Methods) were separated by SDS-PAGE and immunoblotted for the indicated proteins shown on the right.
(TIF)

Figure S5 **Levels of σ^32^ and σ^32^ variants in varying strain backgrounds.** Strains were grown to OD_600_∼0.35, precipitated by addition of TCA to 13% final (vol/vol). Levels of σ^32^ and σ^32^ variants were determined by quantitative immunoblotting (see Materials and Methods). The experiment was carried out ≥5 times, with an example blot shown. These are the raw data used to obtain level values for σ^32^ and its variants shown in Table 3. Averaged quantification of the amount β′ served as a loading control, and levels of FtsH and FtsY are additionally shown. The genetic backgrounds of the mutant strains are shown below the blots. The specific protein probed on each blot is shown to the right. Note that IM-σ^32^ and IM-I54Nσ^32^ run as a smear, most likely because the membrane localization signal adopts multiple conformations during SDS-PAGE electrophoresis. To minimize this problem, gels were run very slowly (60–80 volts). Amount of IM-σ^32^ variants was calculated over the entire smear. Additionally, there is a contaminating band in all samples marked with an asterisk (*) that runs approximately at the same molecular weight as IM-σ^32^. This contaminating band prevents accurate quantification of samples with low amounts of IM-σ^32^ (lanes 2, 7, and 9).
(TIF)

Figure S6 **The 3L-Pf3 peptide does not alter the stability of the FliA σ.** (A) Addition of the 3L-Pf3 peptide to the N-terminus of FliA σ does not affect its cellular levels. Total cellular lysates were separated on SDS-PAGE and immunoblotted for FliA. WT *fliA* or 3L-Pf3-*fliA* was expressed from uninduced pTrc99A in the MG1655 background, and the *fliA* variants expressed are shown (at top). MG1655 carrying only pTrc99A (Vector) shows the endogenous levels of FliA. The lower band present in the 3L-Pf3-FliA lysate is endogenous FliA. Experiments were performed at least three times. The representative experiment shown demonstrates that addition of the 3L-PF3 peptide does not alter the amount of the FliA present in the lysate. As both FliA and 3L-Pf3-FliA are expressed from the same transcriptional and translational start points, we conclude that the 3L-Pf3 tag does not destabilize FliA. Thus, even though targeted to the membrane, 3L-Pf3FliA is not degraded by the membrane localized FtsH protein, which preferentially degrades membrane proteins. (B) Addition of the 3L-Pf3 peptide to the N-terminus of FliA increases its membrane localization. Soluble and membrane fractions from cellular fractionations of MG1655 carrying *fliA* or 3L-Pf3-*fliA* expressed on pTrc99A were separated on SDS-PAGE and immunoblotted for FliA. Percentage of membrane-localized FliA is plotted. Averages of four independent experiments are shown.
(TIF)

Figure S7 **Growth defects in I54Nσ^32^ and p** ***ftsY*** **::Tn** ***5*** **are relieved when the endogenous σ^32^ is membrane-tethered.** (A) Early exponential growth comparison of WT, IM-WTσ^32^, I54Nσ^32^, and IM-I54Nσ^32^. (B) Early exponential growth comparison of WT, IM-WTσ^32^, p*ftsY*::Tn*5* mutant, and the double mutant p*ftsY*::Tn*5*, IM-WTσ^32^. Cellular density (OD_600_) was plotted over time in (A) and (B). Experiments for both (A) and (B) were carried out three times, and an example growth curve obtained is shown. (C) IM-tethering of σ^32^ in mutant strains restores growth rates to that of WT. Doubling times were calculated as the inverse of the slope of the cultures growing in early exponential phase in LB at 30°C. Strain mutations are shown on the left. The exact values of the doubling times for each strain are shown on the right and are an average of three experiments. (D) Membrane-tethering of σ^32^ in the p*ftsY*::Tn*5* mutant restores transition into stationary phase growth to that of WT. The p*ftsY*::Tn*5* mutant transitions into stationary phase growth significantly earlier and at a lower OD_600_ than both WT and the double mutant p*ftsY*::Tn*5*, IM-WTσ^32^. Cellular density (OD_600_) was plotted over time. Growth curves are an average of three biological replicates.
(TIF)

## Abbreviations

HSPs: heat shock proteins
HSR: heat shock response
IM: inner membrane
pBPA: *p*-benzoylphenylalanine
PhoA: alkaline phosphatase
S.A.: specific activity
SR: signal receptor
SRP: signal recognition particle
