# Supplementary figures and images for: Heat Shock Transcription Factor σ32 Co-opts the Signal Recognition Particle to Regulate Protein Homeostasis in E. coli
Source: PLoS Biol. 2013 Dec 17;11(12):e1001735. doi: 10.1371/journal.pbio.1001735 (PMC3866087; doi:10.1371/journal.pbio.1001735)

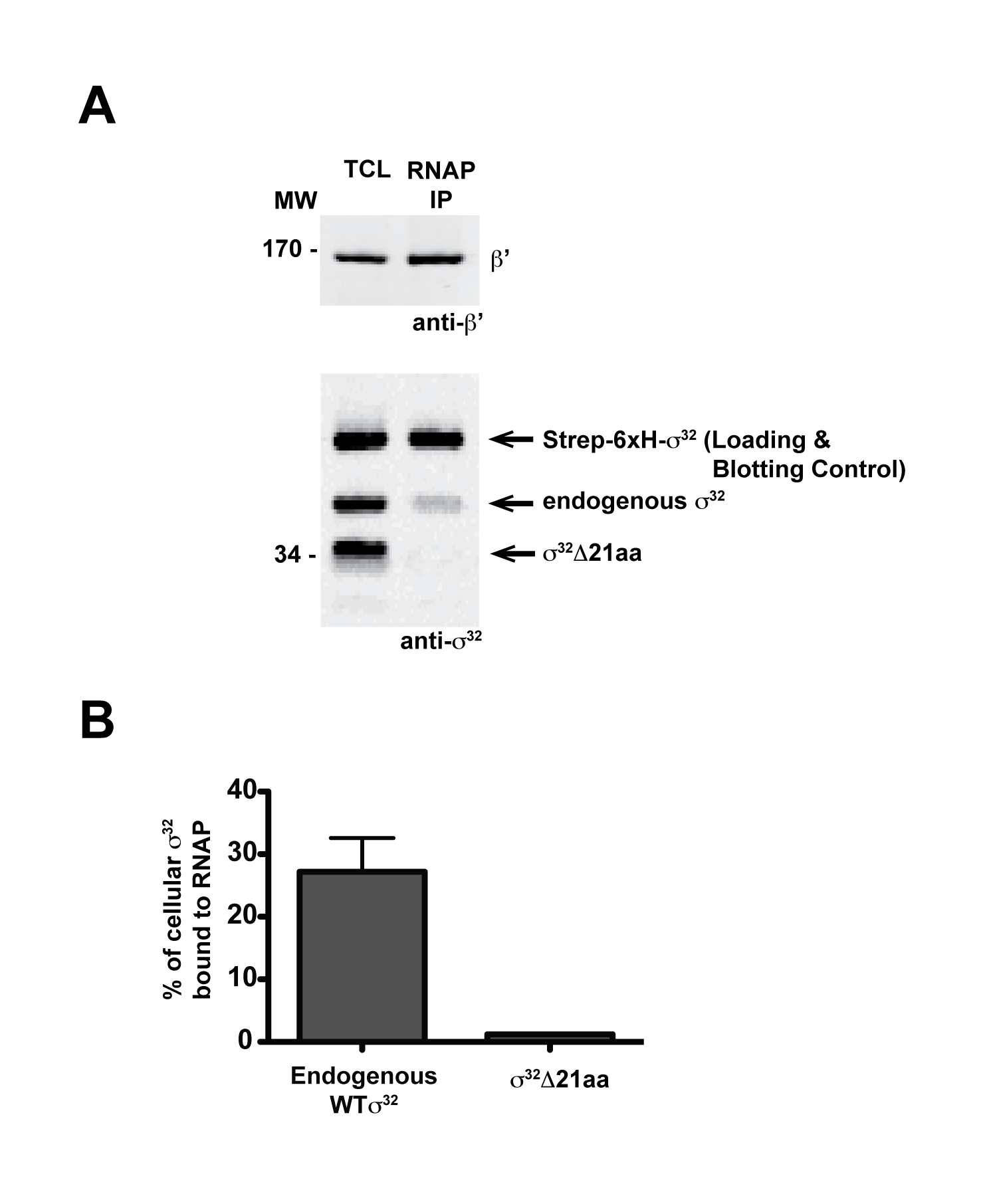

Supplement: Figure S1 — σ32Δ21aa, a C-terminal truncation of σ32, is defective in binding to RNA polymerase in vivo . (A) Immunoprecipitation of RNA polymerase-bound native σ32 and σ32Δ21aa. σ32Δ21aa was expressed from pTrc99A in ΔftsH cells, induced to levels comparable to endogenous σ32, grown to mid-exponential at 30°C in LB medium and the amount of σ32 bound to the anti-β′ resin (Softag4; Neoclone) and remaining σ32 in the supernatant was quantified by immunoblotting using a polyclonal antibody against σ32. Comparable amounts of total cellular lysates (TCL; left lane) and corresponding RNA-polymerase immunoprecipitations (RNAP IP; right lane) are shown. Purified σ32, tagged at the N-terminus with a Strep and 6×Histidine (Strep-6×H) tag, was used as a loading and blotting control. Results of a representative experiment are shown. (B) Quantification of RNA polymerase-bound native σ32 and σ32Δ21aa expressed in the same strain background (ΔftsH). Averages of four independent experiments are shown. (TIF) [file pbio.1001735.s001.tif]

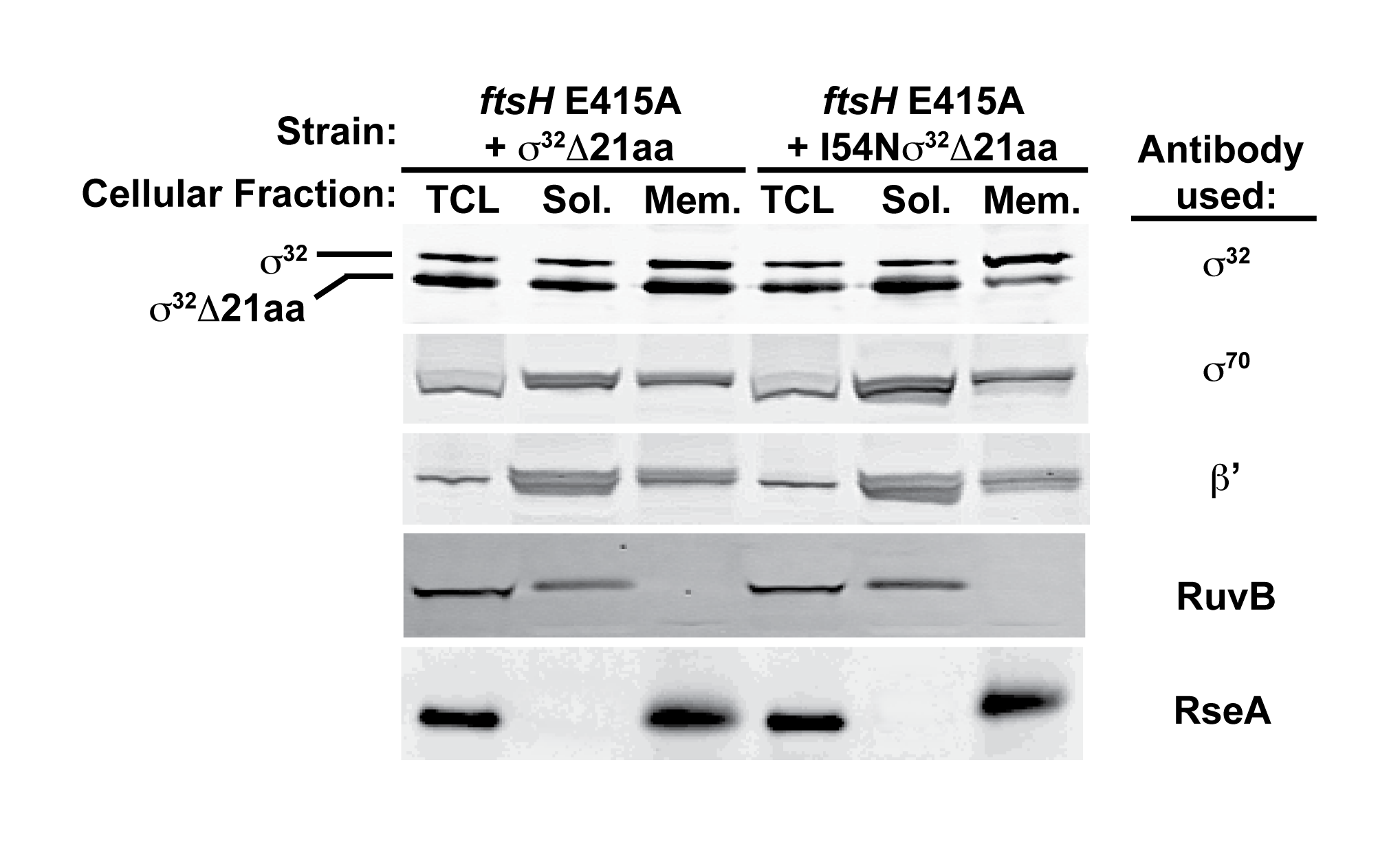

Supplement: Figure S2 — Membrane fractionation of σ32 is independent of RNA polymerase binding. ftsH E415A cells expressing either WTσ32Δ21aa or I54Nσ32Δ21aa were subjected to cellular fractionation (see Materials and Methods), and soluble and membrane fractions were resolved by SDS-PAGE and analyzed by immunoblotting for σ32, σ70, and the β′ subunit of RNA polymerase. Ectopically expressed σ32Δ21aa or I54Nσ32Δ21aa were present at levels comparable to native σ32 and were distinguished from endogenous σ32 on a 10% SDS-PAGE gel. All fractionation experiments were performed ≥8 times, and % fractionation was calculated from experiments where probed cytoplasmic (RuvB) and membrane (RseA) proteins separated properly. (TIF) [file pbio.1001735.s002.tif]

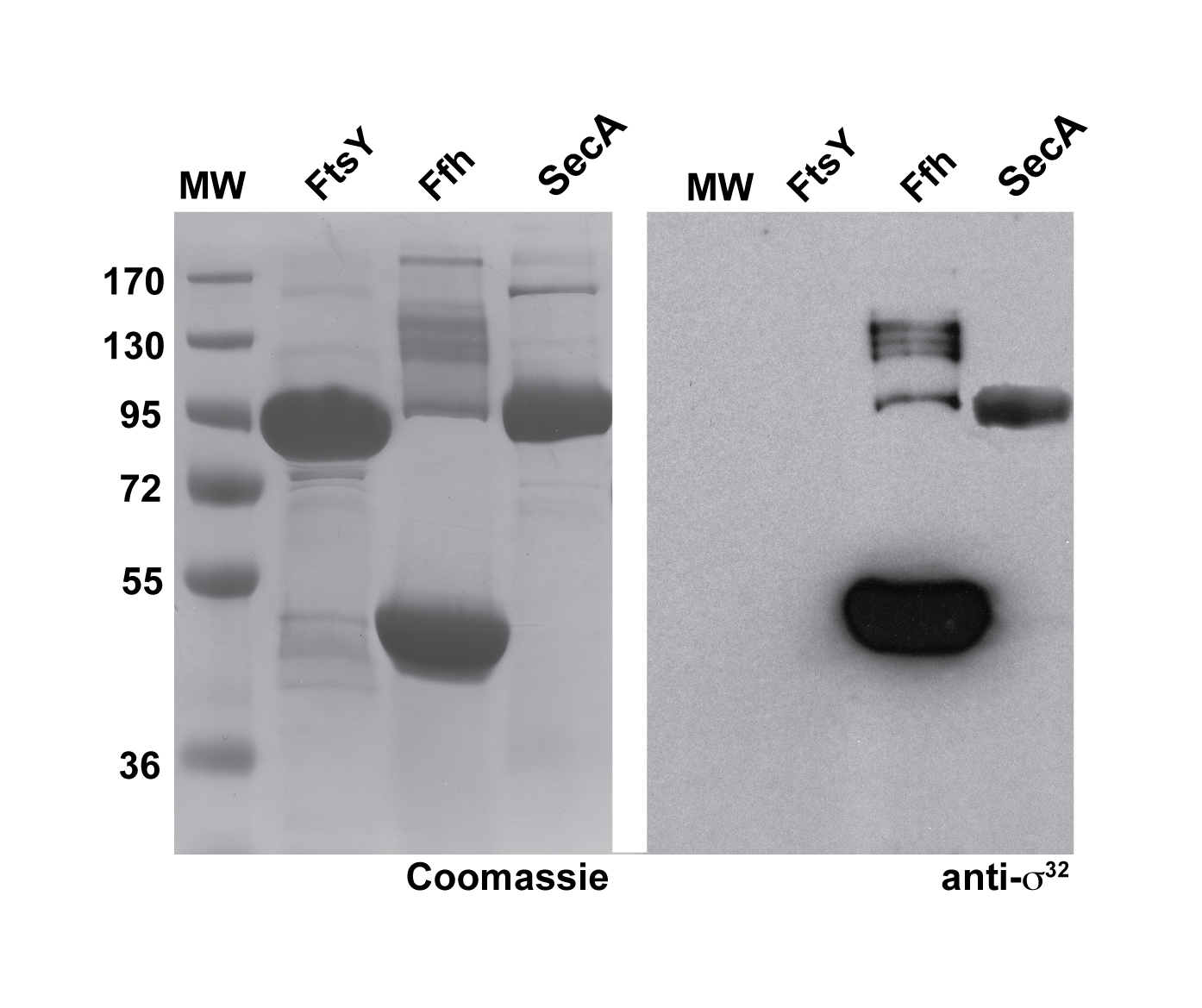

Supplement: Figure S3 — σ32 interacts with SecA through protein–protein interaction analysis. Purified SecA was run on a 10% SDS-PAGE gel (along with FtsY and Ffh), transferred to nitrocellulose, re-natured, and incubated with purified WTσ32. The Coomassie-stained gel of the prey proteins (FtsY, Ffh, and SecA; left) and the nitrocellulose membrane containing the transferred prey proteins, probed with polyclonal anti-σ32 antibodies (right), are shown. The Coomassie-stained gel section of FtsY and Ffh and the corresponding σ32-incubated nitrocellulose membrane probed with anti-σ32 antibodies are also shown in Figure 2C. (TIF) [file pbio.1001735.s003.tif]

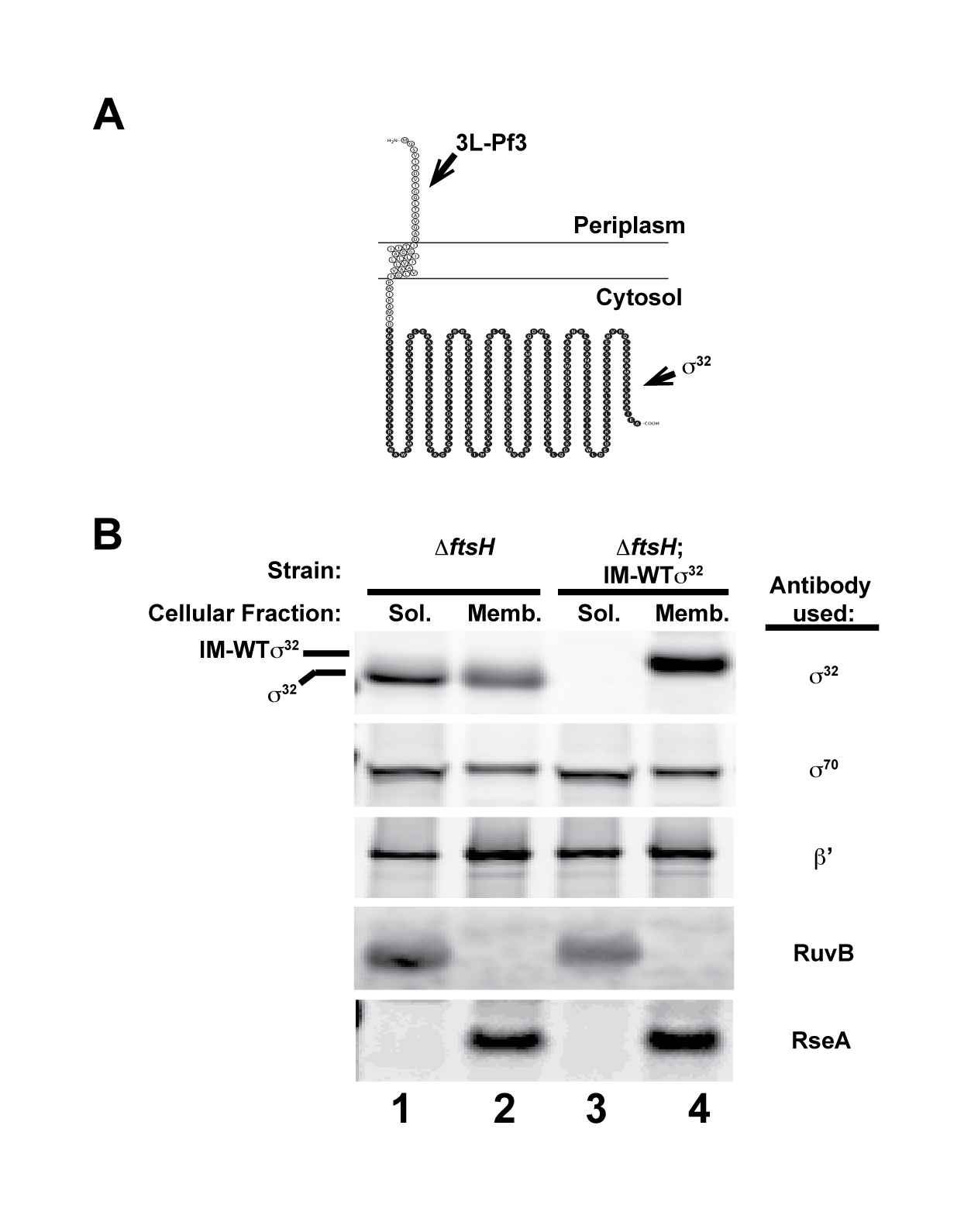

Supplement: Figure S4 — Fusing the 3L-Pf3 peptide to the N-terminus of WTσ32 coding sequence significantly increases its membrane localization. (A) Schematic representation of membrane-tethered 3L-Pf3-WTσ32 (IM-WTσ32). The amino acids corresponding to the 3L-Pf3 and σ32 are shown as open or enclosed dark circles, respectively. (B) Soluble (lanes 1 and 3) and membrane (lanes 2 and 4) fractions from cellular fractionations (described in Materials and Methods) were separated by SDS-PAGE and immunoblotted for the indicated proteins shown on the right. (TIF) [file pbio.1001735.s004.tif]

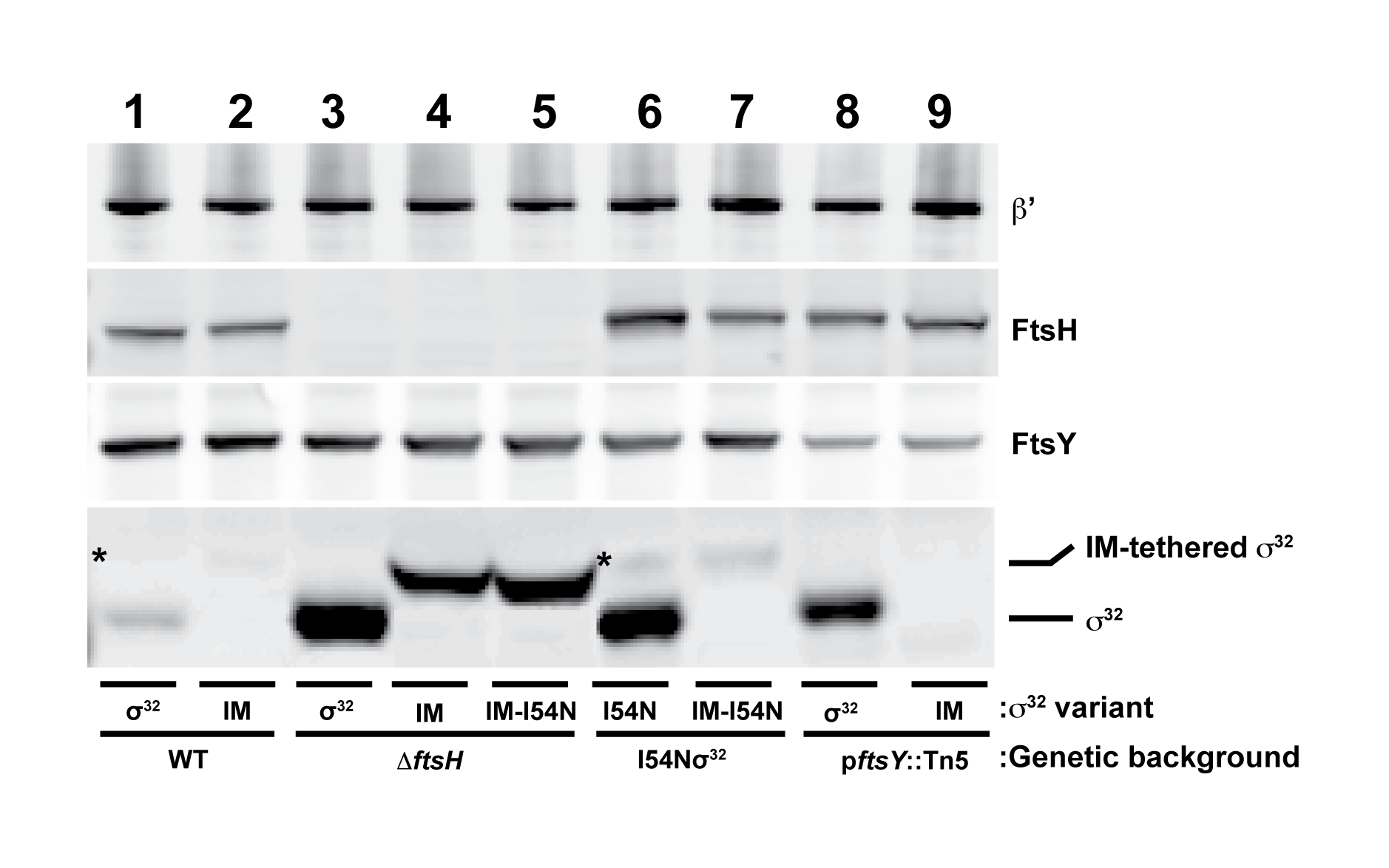

Supplement: Figure S5 — Levels of σ32 and σ32 variants in varying strain backgrounds. Strains were grown to OD600∼0.35, precipitated by addition of TCA to 13% final (vol/vol). Levels of σ32 and σ32 variants were determined by quantitative immunoblotting (see Materials and Methods). The experiment was carried out ≥5 times, with an example blot shown. These are the raw data used to obtain level values for σ32 and its variants shown in Table 3. Averaged quantification of the amount β′ served as a loading control, and levels of FtsH and FtsY are additionally shown. The genetic backgrounds of the mutant strains are shown below the blots. The specific protein probed on each blot is shown to the right. Note that IM-σ32 and IM-I54Nσ32 run as a smear, most likely because the membrane localization signal adopts multiple conformations during SDS-PAGE electrophoresis. To minimize this problem, gels were run very slowly (60–80 volts). Amount of IM-σ32 variants was calculated over the entire smear. Additionally, there is a contaminating band in all samples marked with an asterisk (*) that runs approximately at the same molecular weight as IM-σ32. This contaminating band prevents accurate quantification of samples with low amounts of IM-σ32 (lanes 2, 7, and 9). (TIF) [file pbio.1001735.s005.tif]

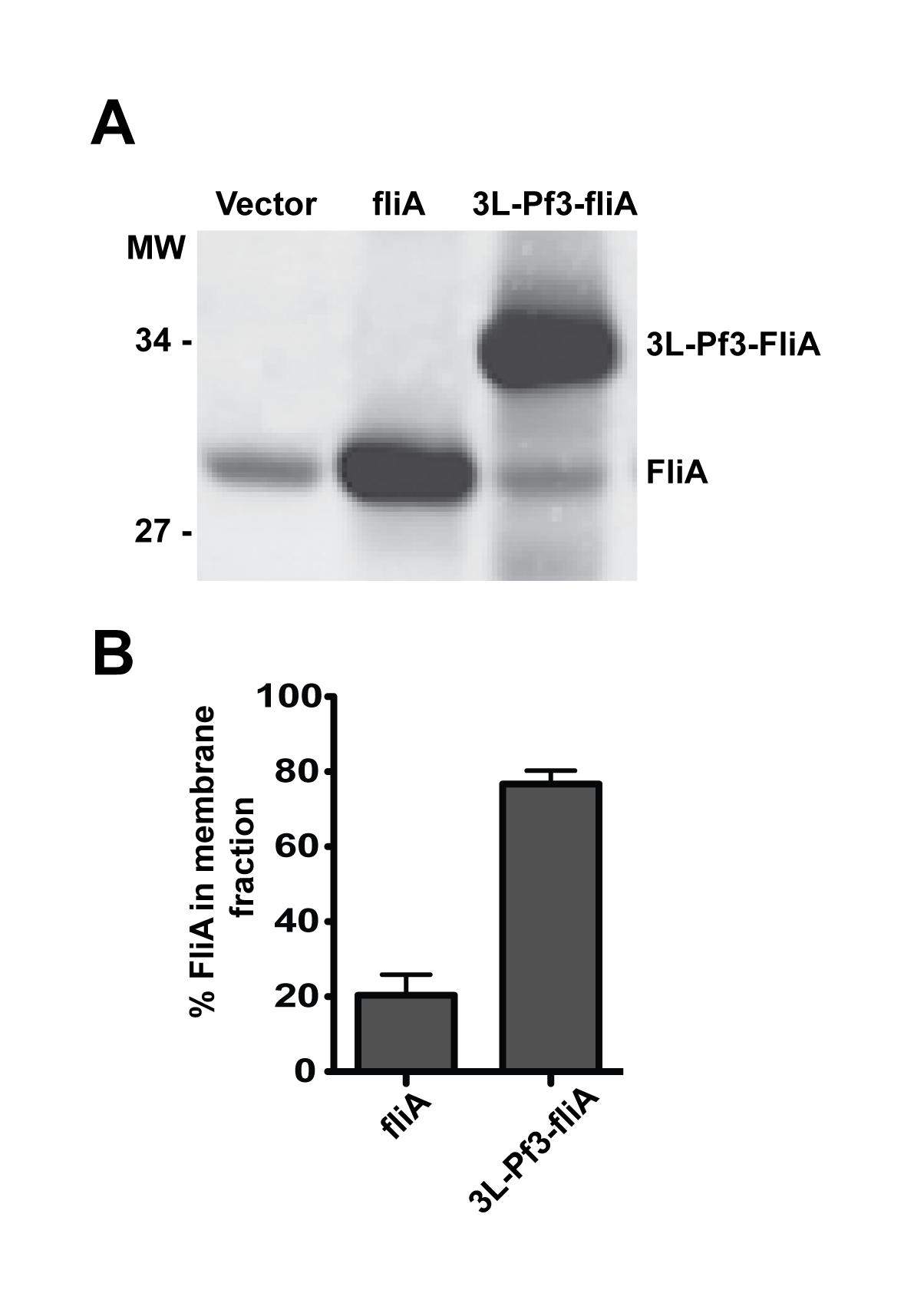

Supplement: Figure S6 — The 3L-Pf3 peptide does not alter the stability of the FliA σ. (A) Addition of the 3L-Pf3 peptide to the N-terminus of FliA σ does not affect its cellular levels. Total cellular lysates were separated on SDS-PAGE and immunoblotted for FliA. WT fliA or 3L-Pf3-fliA was expressed from uninduced pTrc99A in the MG1655 background, and the fliA variants expressed are shown (at top). MG1655 carrying only pTrc99A (Vector) shows the endogenous levels of FliA. The lower band present in the 3L-Pf3-FliA lysate is endogenous FliA. Experiments were performed at least three times. The representative experiment shown demonstrates that addition of the 3L-PF3 peptide does not alter the amount of the FliA present in the lysate. As both FliA and 3L-Pf3-FliA are expressed from the same transcriptional and translational start points, we conclude that the 3L-Pf3 tag does not destabilize FliA. Thus, even though targeted to the membrane, 3L-Pf3FliA is not degraded by the membrane localized FtsH protein, which preferentially degrades membrane proteins. (B) Addition of the 3L-Pf3 peptide to the N-terminus of FliA increases its membrane localization. Soluble and membrane fractions from cellular fractionations of MG1655 carrying fliA or 3L-Pf3-fliA expressed on pTrc99A were separated on SDS-PAGE and immunoblotted for FliA. Percentage of membrane-localized FliA is plotted. Averages of four independent experiments are shown. (TIF) [file pbio.1001735.s006.tif]

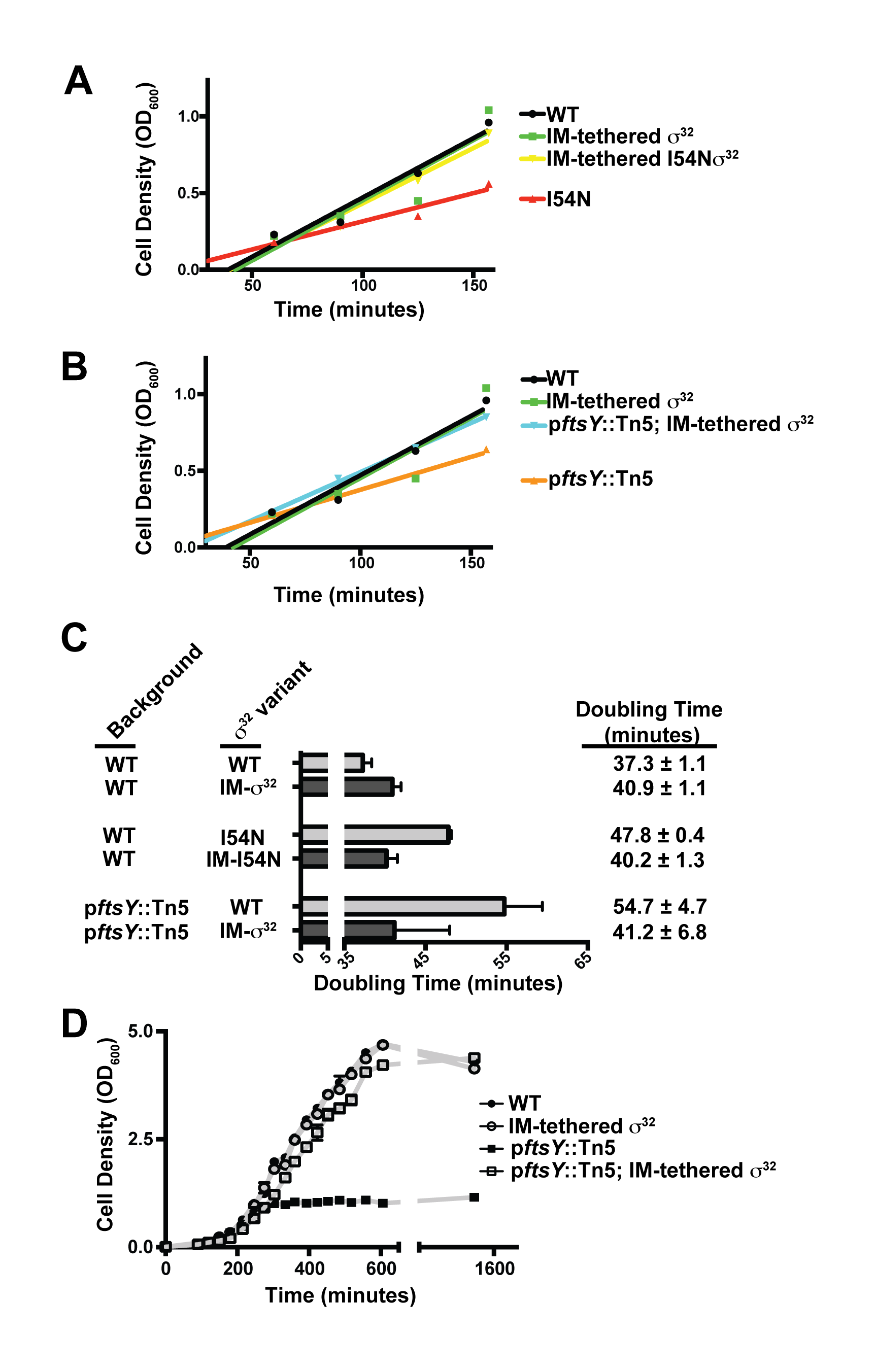

Supplement: Figure S7 — Growth defects in I54Nσ32 and p ftsY ::Tn 5 are relieved when the endogenous σ32 is membrane-tethered. (A) Early exponential growth comparison of WT, IM-WTσ32, I54Nσ32, and IM-I54Nσ32. (B) Early exponential growth comparison of WT, IM-WTσ32, pftsY::Tn5 mutant, and the double mutant pftsY::Tn5, IM-WTσ32. Cellular density (OD600) was plotted over time in (A) and (B). Experiments for both (A) and (B) were carried out three times, and an example growth curve obtained is shown. (C) IM-tethering of σ32 in mutant strains restores growth rates to that of WT. Doubling times were calculated as the inverse of the slope of the cultures growing in early exponential phase in LB at 30°C. Strain mutations are shown on the left. The exact values of the doubling times for each strain are shown on the right and are an average of three experiments. (D) Membrane-tethering of σ32 in the pftsY::Tn5 mutant restores transition into stationary phase growth to that of WT. The pftsY::Tn5 mutant transitions into stationary phase growth significantly earlier and at a lower OD600 than both WT and the double mutant pftsY::Tn5, IM-WTσ32. Cellular density (OD600) was plotted over time. Growth curves are an average of three biological replicates. (TIF) [file pbio.1001735.s007.tif]
